# Patulin suppresses α_1_-adrenergic receptor expression in HEK293 cells

**DOI:** 10.1038/s41598-020-77157-0

**Published:** 2020-11-18

**Authors:** Yashodani Pillay, Savania Nagiah, Alisa Phulukdaree, Anand Krishnan, Anil A. Chuturgoon

**Affiliations:** 1 Discipline of Medical Biochemistry, University of KwaZulu-Natal, Durban, South Africa; 2 Discipline of Medical Biochemistry and Chemical Pathology, Faculty of Health Sciences, Howard College, University of KwaZulu-Natal, George Campbell Building, Durban, 4041 South Africa; 3 Present Address: Department of Physiology, Nelson Mandela Metropolitan University, Port Elizabeth, South Africa; 4 Present Address: Department of Physiology, University of Pretoria, Pretoria, South Africa; 5 Present Address: Department of Chemical Pathology, University of Free State, Bloemfontein, South Africa

**Keywords:** Biochemistry, Cell biology, Computational biology and bioinformatics, Molecular biology, Nephrology, Risk factors

## Abstract

Patulin (PAT) is a common mycotoxin contaminant of apple products linked to impaired metabolic and kidney function. Adenosine monophosphate activated protein kinase (AMPK), abundantly expressed in the kidney, intercedes metabolic changes and renal injury. The alpha-1-adrenergic receptors (α_1_-AR) facilitate Epinephrine (Epi)-mediated AMPK activation, linking metabolism and kidney function. Preliminary molecular docking experiments examined potential interactions and AMPK-gamma subunit 3 (PRKAG3). The effect of PAT exposure (0.2–2.5 µM; 24 h) on the AMPK pathway and α_1_-AR was then investigated in HEK293 human kidney cells. AMPK agonist Epi determined direct effects on the α_1_-AR, metformin was used as an activator for AMPK, while buthionine sulphoximine (BSO) and *N*-acetyl cysteine (NAC) assessed GSH inhibition and supplementation respectively. *ADRA1A* and *ADRA1D* expression was determined by qPCR. α_1_-AR, ERK1/2/MAPK and PI3K/Akt protein expression was assessed using western blotting. PAT (1 µM) decreased α_1_-AR protein and mRNA and altered downstream signalling. This was consistent in cells stimulated with Epi and metformin. BSO potentiated the observed effect on α_1_-AR while NAC ameliorated these effects. Molecular docking studies performed on Human ADRA1A and PRKAG3 indicated direct interactions with PAT. This study is the first to show PAT modulates the AMPK pathway and α_1_-AR, supporting a mechanism of kidney injury.

## Introduction

Patulin (PAT) is a mycotoxin produced by *Penicillium, Bissochlamys* and *Aspergillus* sp.^1^. These moulds contaminate overripe, rotting apples and apple products. A safety level of 50 μg/l PAT in consumables was established following mounting evidence of adverse effects in exposed humans and animals^2,3^. Despite this regulation there are vast variations in PAT concentrations in apple products worldwide^2^.

On a molecular level, PAT exerts toxicity by covalently binding to thiol groups in proteins and forming adducts with DNA^4,5^. This action depletes cellular antioxidant glutathione (GSH), contributing to oxidative stress, compromised mitochondrial function, decreased ATP production and cell death^6–9^. These deleterious effects are most commonly observed in the kidney, gastrointestinal tract, liver and brain of PAT exposed subjects^3,10,11^.

The kidneys have several properties to support its functional role in the metabolism and removal toxins from circulation^12^. This includes high renal blood flow, metabolic demands and the concentrating capacity of the nephron; which also increases susceptibility to toxic insult^13^. ATP is an essential energy source in renal tubules and according to recent studies, PAT exposure compromises cellular ATP levels^11^.

The adenosine monophosphate-activated kinase (AMPK) complex is part of a sophisticated system that enables the cell to alter metabolism based on nutrient availability. AMPK is a cellular energy sensor activated in response to the changes in the ATP:ADP ratio under low energy conditions. AMPK phosphorylates enzymes and influences growth points to positively regulate ATP-generating pathways and decrease ATP consumption^14^. AMPK activation is facilitated by adrenergic receptor signalling. The adrenergic receptors are a group of G protein coupled receptors (GPCR) that mediate the effects of catecholamines; epinephrine (Epi) and norepinephrine. The receptors are classified into three major types; alpha-1 (α_1_-AR), alpha-2 (α_2_-AR) and beta (β-AR). These groups are further divided into subtypes; α-1A, α-1B, α-1D; α-2A, -2B, α-2C and β-1, β-2, β-3 according to their pharmacologic properties^15^.

The α_1_-AR are abundantly expressed in the kidney where they mediate several important functions including metabolism, renal tone and tubule function. Persistent suppression of α_1_-AR and AMPK in the kidney is associated with renal injury and fibrosis^16–18^. Altered renal tone, nephrotoxicity and metabolism are mediated by adrenergic receptor signalling. This indicates α_1_-AR is a potential target for PAT-induced renal toxicity given previously observed effects of PAT on the kidney^19–21^.

α_1_-AR uses a variety of secondary messengers to mediate cellular functions^22^. Activation of α_1_-AR triggers a conformational change facilitating interactions with, and activation of the G_q/11_ protein family and phospholipase C (PLC). PLC hydrolyses phosphatidylinositol 1,2-biphosphate (PIP_2_) to inositol triphosphate (IP_3_) and diacylglycerol (DAG), mobilizing the release of intracellular calcium (Ca^2+^)^23^. Ca^2+^ and DAG then execute cellular functions like smooth muscle contraction and activate protein kinase C (PKC) to phosphorylate cellular proteins that mediate an appropriate cellular response. This includes p21-ras, phosphatidylinositide (PI) 3-kinase (PI3K) and mitogen activated protein kinases (MAPK)^15,22,24^.

α_1_-AR regulated growth responses are coordinated via the MAPK family comprising the extracellular signal-regulated kinases (ERK), c-Jun N-terminal kinases (JNK) and p38 kinases. The activity of the MAPKs is regulated through a series of phosphorylation events which facilitate MAPK-mediated transcription factor and cytosolic protein phosphorylation. The ERK1/2 pathway can be activated by G_q/11_ mediated interactions with Ras and p21 or via direct activation through PKC and Ca^2+^. This action potentially contributes to α_1_-AR mediated DNA synthesis and cell growth^25,26^.

PI3K is functionally positioned downstream of Ras and converts PIP_2_ to phosphatidylinositol (3,4,5)-phosphate (PIP_3_). This results in membrane localisation and phosphorylation of Akt. Active Akt phosphorylates transcription factors and proteins that regulate cell survival and metabolism^15,20,22,27,28^; while Akt suppression is associated with cell death and apoptosis via mitochondrial dysfunction^29,30^.

The MAPK and Akt pathways are redox sensitive signalling proteins critical to cell survival and function. Previous studies show ROS mediates PAT-induced changes in ERK signalling^1^. Another study found ERK and Akt pathways have been implicated in cell survival and carcinogenesis in PAT-exposed murine keratinocytes^31^. Stress response mechanisms determining cell death or survival are central modulators in recovery and progression of renal injury^32^. Evidence for molecular mechanisms of PAT-induced nephrotoxicity in humans remains limited with no available data to date on the α_1_-AR system. This study investigated the effects of PAT on the α_1_-AR and downstream signalling in human embryonic kidney cells (HEK293).

## Results

### PAT binds AMP-activated protein kinase gamma subunit 3 (PRKAG3) with high affinity via molecular docking studies

**Figure 1:**
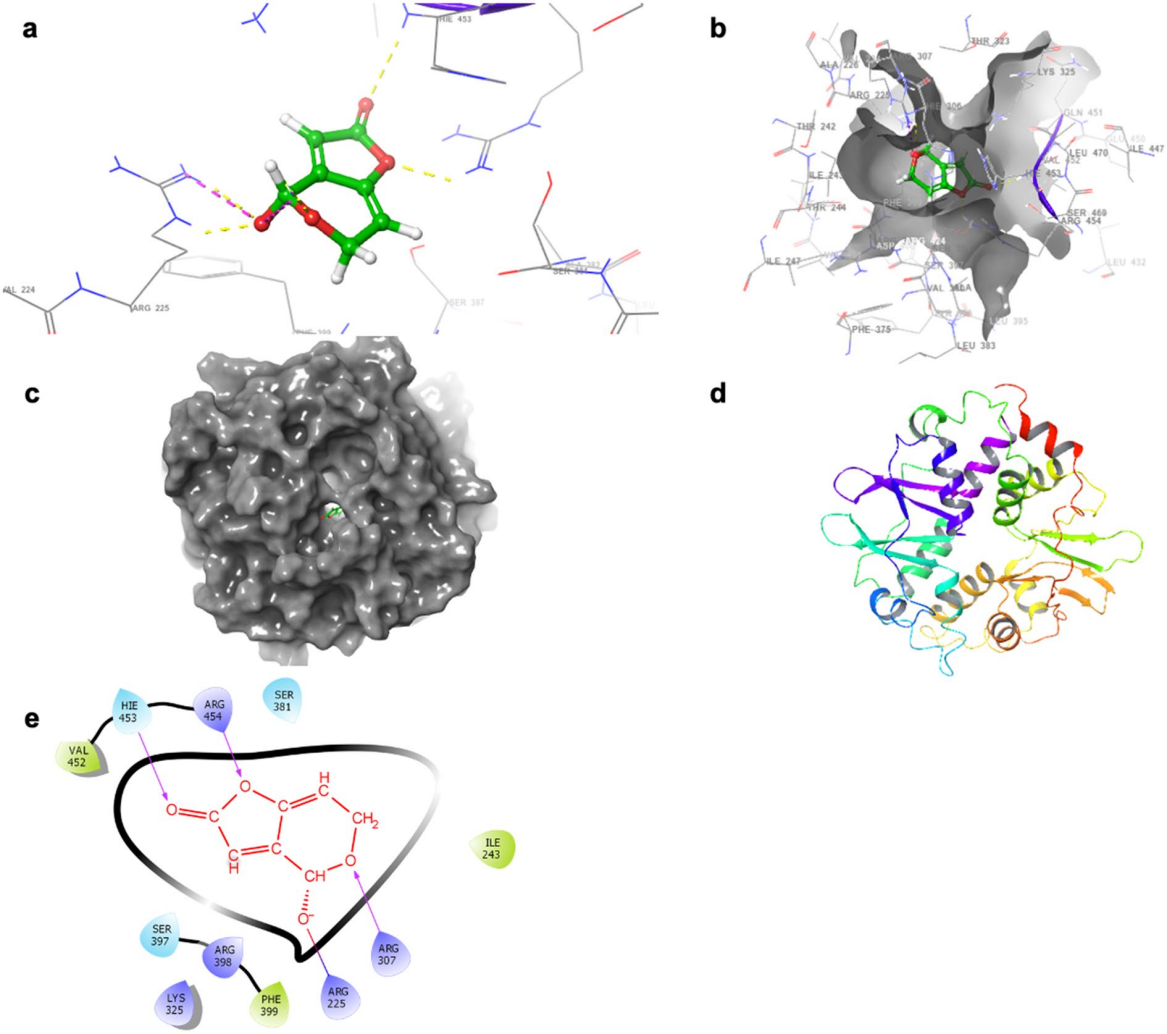
PAT has a high affinity for the AMPK subunit PRKAG3. (**a**,**b**) Space filling 3D model demonstrating hydrogen bonding between PRKAG3 and PAT. (**c**,**d**) indicate PRKAG3 binding pocket and ligand interaction with PAT using a 3D ribbon model while (**e**) shows 2D binding sites of PRKAG3 with PAT.

While it is known that PAT increases oxidative stress and impairs mitochondrial function (Supplementary Data S2), little is known about the effects of PAT on AMPK. Preliminary docking studies of PAT were performed using a theoretically modeled structure of the PRKAG3 protein as a template. The structure of the selective receptor protein active binding site was chosen based on the active domain reported in previous studies. The identified active site amino acid based binding pocket grid was created for further analysis. The results revealed molecular interactions which account for the observed affinity within 4 Å distance (Fig. 1). The hydroxyl groups of the myricetin interacted through hydrogen bonding with the side chain residues of ARG307, ARG454, and HIE453 on the PRKAG3 receptor (Fig. 1). The binding efficiency of PAT to PRKAG3 was relatively strong with an estimated affinity of − 5.734 kcal/mol. Both docked complexes were examined with an emphasis on visual rather than numerical appraisal, so XP were used for further results presented.

### Cytotoxic levels of PAT alter α-adrenergic receptor expression in HEK293 cells

**Figure 2:**
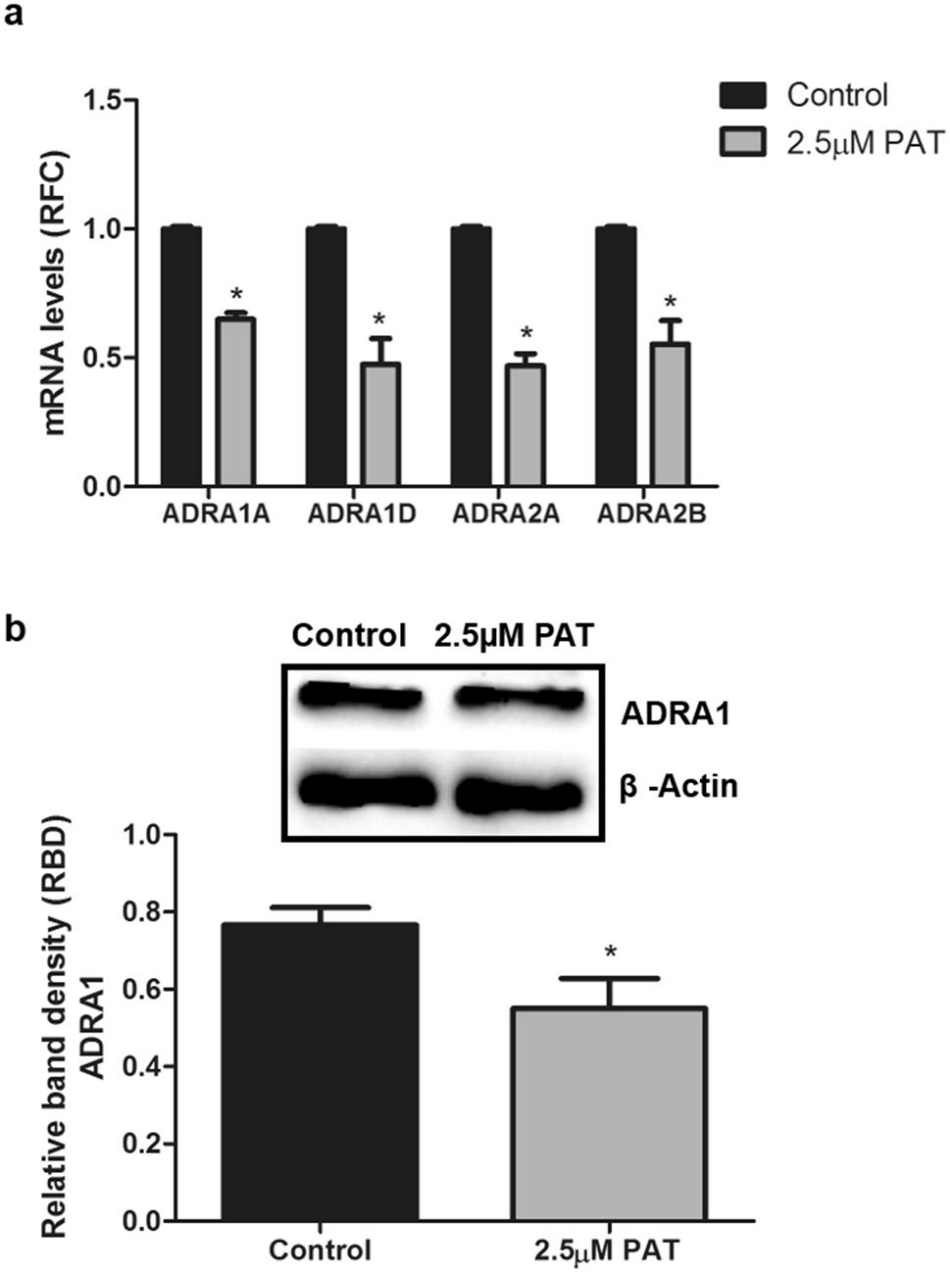
PAT alters α-adrenergic receptor expression at both transcriptional and translational levels in HEK293 cells. Decreased α_1_ and α_2_ adrenergic receptor (*ADRA1A, ADRA1D, ADRA2A, ADRA2B*). (**a**) Relative fold change (RFC) in mRNA levels by 2.5 µM PAT were validated by qPCR (p = 0.0294). (**b**) Decreased protein expression of α_1_-AR was confirmed using western blotting (p = 0.0286) (*p < 0.05 relative to untreated control). The original western blot presented here is available in Supplementary Data S5.

PAT compromises ATP production and mitochondrial health^11^ (Supplementary Data S2) and binds PRKAG3 with high affinity (Fig. 1). Hence, the effects of 2.5 µM PAT^33,34^ on AMPK signalling gene expression was measured using a Super Array Profiler of 84 relevant genes. Fold change > 2 was considered significant (Full array results listed in Supplementary Data S3). It was determined 2.5 µM PAT differentially regulated α-adrenergic receptor genes (Table 1; *ADRA1A, ADRA1D, ADRA2A, ADRA2B*) in this pathway. Findings were corroborated by RT-PCR that showed PAT significantly decreased the *ADRA1A, ADRA1D, ADRA2A* and *ADRA2B* receptor subtype mRNA levels (p = 0.0294; Fig. 2a). Western blotting further validated findings showing α_1_-AR protein expression was significantly decreased (1.4-fold; p = 0.0286; Fig. 2b) by PAT.

**Table 1:** Human AMPK signalling PCR Array showing significantly differentially regulated α-adrenergic receptor gene expression.

Gene	Description	Fold change
*ADRA1A*	Alpha-1A adrenergic receptor	− 2.26
*ADRA1D*	Alpha-1D adrenergic receptor	− 2.52
*ADRA2A*	Alpha-2A adrenergic receptor	− 2.66
*ADRA2B*	Alpha-2B adrenergic receptor	− 3.91

*Fold change > 2 was considered significant, full array results are listed in Supplementary Data S3.

### Environmental PAT exposure alters α-adrenergic receptor expression in HEK293 cells

**Figure 3:**
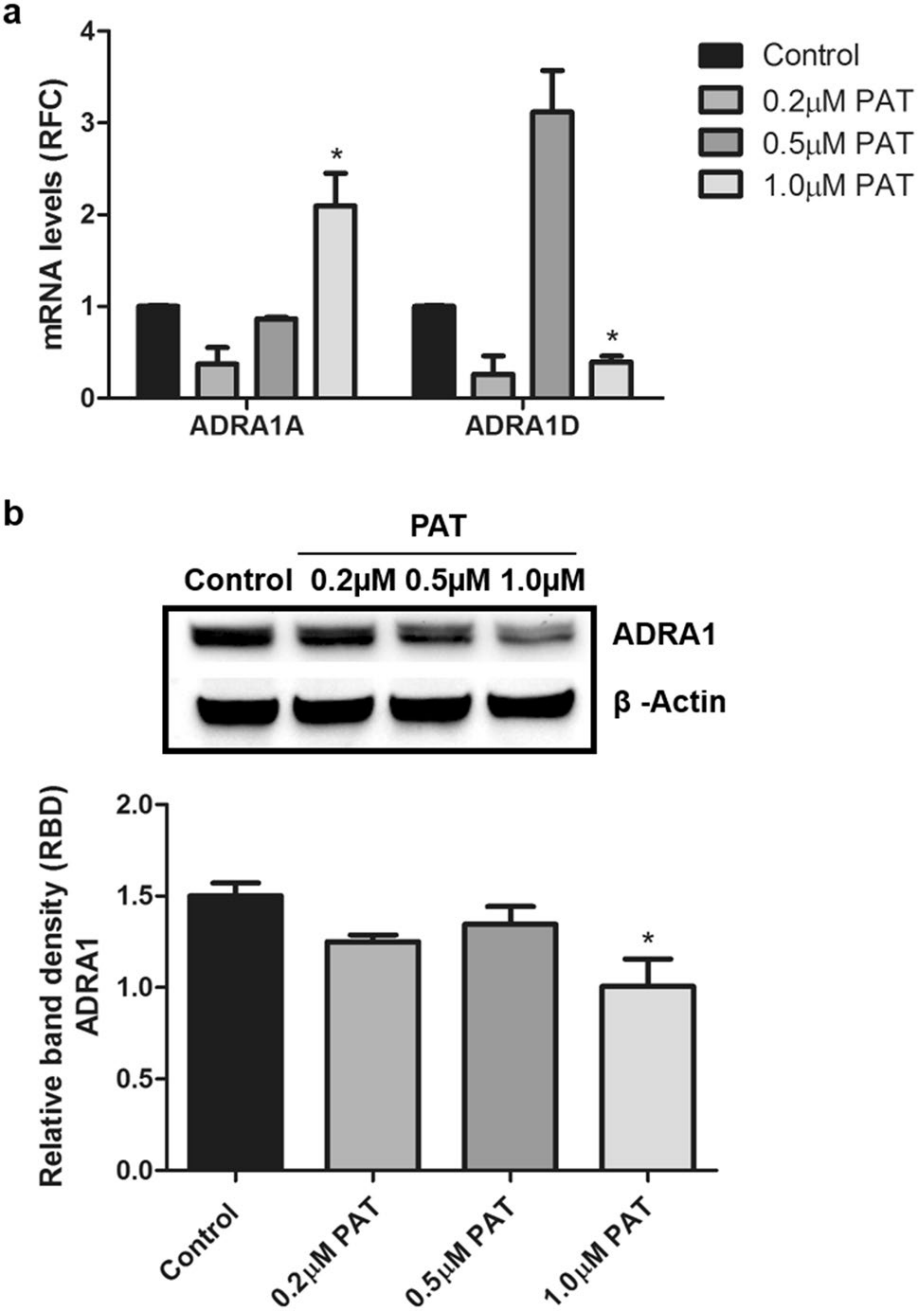
Environmental levels of PAT alter transcript and protein α_1_-AR expression levels. HEK293 cells were treated with PAT (0.2 µM; 0.5 µM; 1 µM) for 24 h. (**a**) Using qPCR, PAT significantly altered relative fold change (RFC) *ADRA1A* (p = 0.0165) and *ADRA1D* (p = 0.0043) mRNA levels and (**b**) significantly decreased α_1_-AR protein expression at all concentrations (p = 0.0037) (*p < 0.05 relative to untreated control) The original western blot presented is available in Supplementary Data S6.

α_1_-AR expression was determined in HEK293 cells exposed to PAT (0.2 µM; 0.5 µM, 1.0 µM) concentrations relevant to environmental exposure; including safety levels and concentrations recorded in incidence, evaluation and consumer studies^3,35^. As shown in Fig. 3; significant changes to *ADRA1A* (p = 0.0043; Fig. 3a) and *ADRA1D* (p = 0.0165; Fig. 3a) mRNA levels were observed after 24 h exposure. Measures of α_1_-AR protein expression (which was inclusive of all ADRA1 subtypes) was determined to have decreased consistently across treatments, most notably at 1 µM (p = 0.0037; Fig. 3b). An observed threefold increase in *ADRA1D* was noted at 0.5 μM. However, the relatively high SD prevented statistical significance.

### PAT decreases α_1_-adrenergic receptor protein expression and opposes Epi and AMPK action

**Figure 4:**
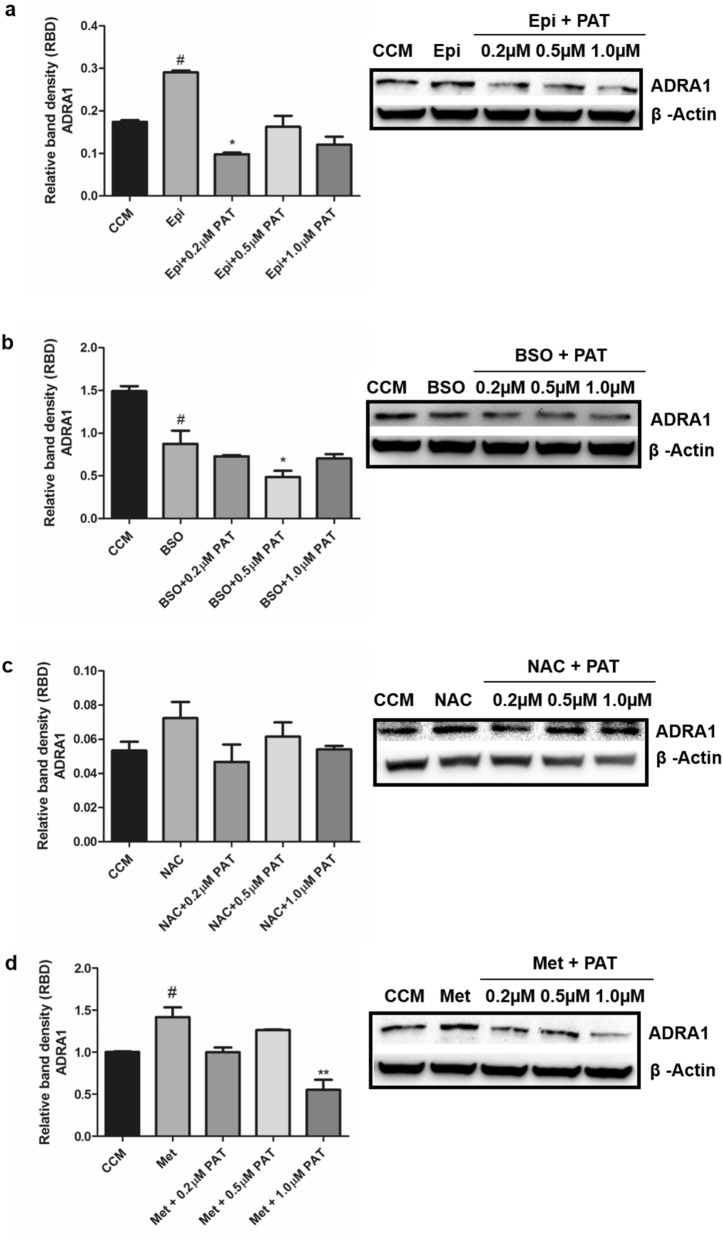
PAT alters α_1_-adrenergic receptor protein expression in HEK293 cells. Western blotting determined (**a**) α_1_-AR expression decreased significantly following PAT administration in cells pre-exposed to α_1-_AR agonist Epi (p = 0.0145). GSH depletion mimicked by (**b**) BSO (p = 0.0121) decreased expression significantly; while supplementation by (**c**) NAC (p = 0.0941) showed no changes before and after PAT exposure however, (**d**) metformin-exposed cells showed a significant increase in ADRA1 (p = 0.0294) and a significant decrease (p = 0.001) following PAT exposure (^#^*p* < 0.05 relative to untreated control; *p < 0.05 relative to respective pre-treated control) The original western blots are presented in Supplementary Data S7.

The effects of PAT on α_1_-AR were assessed using pathway agonist, Epi. In Epi-stimulated cells, α_1_-AR protein expression was elevated relative to standard culture conditions (p = 0.0265). Following PAT addition however, protein expression decreased significantly across all concentrations, most potently at 0.2 µM and 1 µM (p = 0.0145; Fig. 4a).

BSO and NAC mimic and oppose PAT actions on GSH respectively. BSO was used to determine whether PAT-related thiol depletion had altered the α_1_-AR expression. In these treatments α_1_-AR protein expression was decreased compared to controls (p = 0.0286). The decrease was further exacerbated following PAT treatments (p = 0.0121; Fig. 4b). NAC, a GSH precursor was used to assess whether these effects could be reversed. Findings showed no change to α_1_-AR protein expression in PAT treatments (p = 0.0941; Fig. 4c) in cells pre-exposed to NAC.

Metformin, a mitochondrial inhibitor and AMPK activator was used to assess the effects of PAT on α_1_-AR via the AMPK pathway. It was determined metformin caused a significant increase in α_1_-AR protein expression (p = 0.0294; Fig. 4d). PAT addition however significantly decreased α_1_-AR expression (p = 0.001; Fig. 4d), most significantly at 1 µM—above the established safety level.

These results allude to an inhibitory role for PAT in Epi-mediated α_1_-AR signalling and AMPK signalling with GSH depletion as a possible mitigating factor (Fig. 4).

### PAT binds ADRA1 with strong affinity via computer docking studies

**Figure 5:**
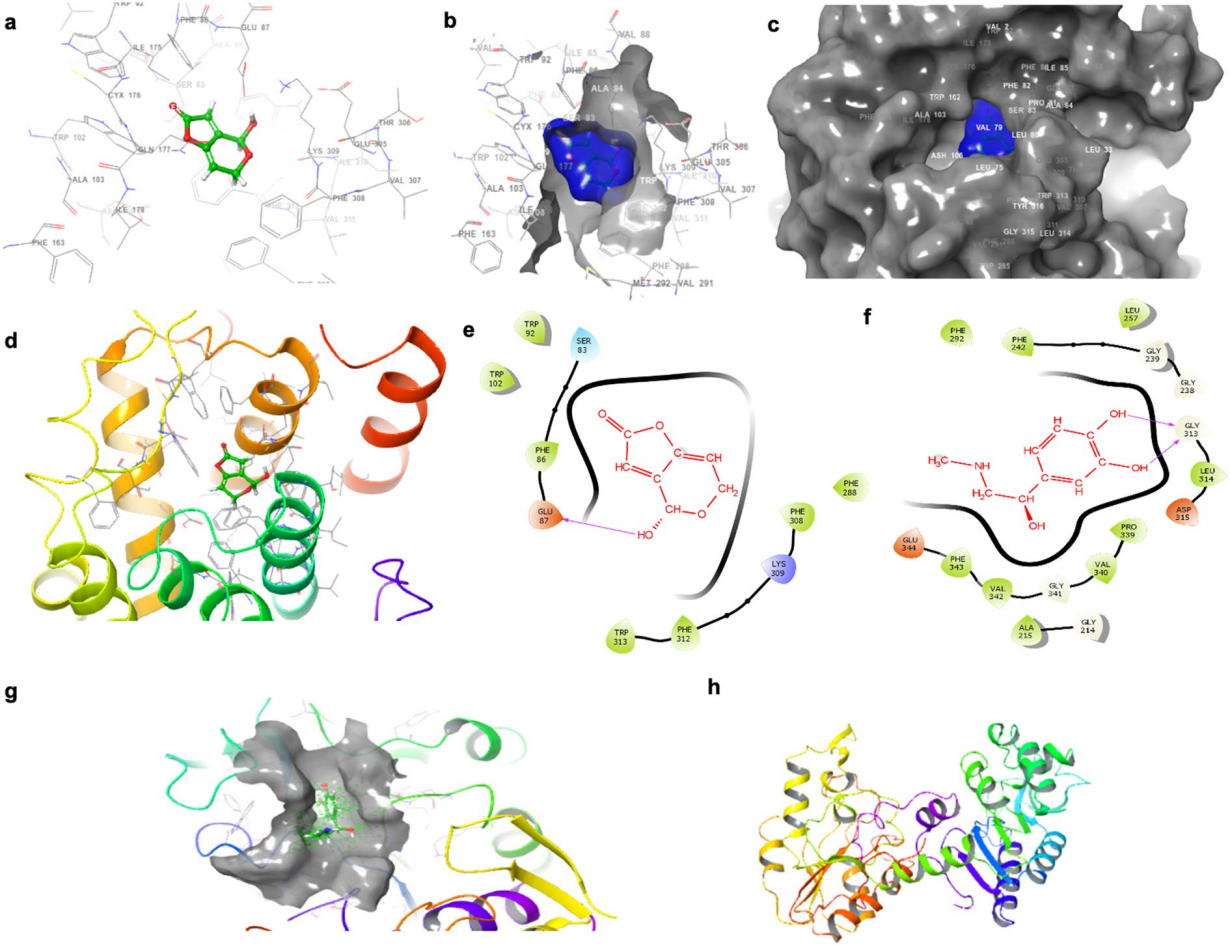
Molecular docking determined PAT has a high affinity for the ADRA1 receptor with an alternate binding site to Epi. (**a**,**b**) Space filling 3D indicating hydrogen bonding between ADRA1 and PAT. (**c**,**d**) 3D Ribbon model of ADRA1 and PAT demonstrating the binding pocket and ligand interaction profile. (**e**) 2D binding sites of ADRA1 and PAT. (**f**) 2D binding sites of ADRA1 and Epi. (**g**) Space filling 3D indicating binding between Epi and ADRA1. (**h**) 3D Ribbon model showing the binding pocket and ligand interaction profile of Epi and ADRA1.

Computational methods were used to check the affinity binding between PAT and ADRA1, and Epi and ADRA1. Molecular docking predicts the binding modes and affinities of ligands and their receptors. An analysis of the docked complex of ADRA1 revealed highly significant interactions between PAT (ligand) and the ADRA1 receptor. Both 3D and 2D images were generated using Modeler software to visualise the interaction between PAT and ADRA1 (Fig. 5a,b). The total free energy of binding was estimated to be − 6.4 kcal/mol, suggestive of a favourable reaction. PAT readily bound and formed close interactions with residues of ADRA1 through a variety of interactions including hydrogen bonding, pi–pi bonding, pi–alky bonding (Fig. 5c,d). The binding efficiency of PAT to ADRA1 was relatively strong with an estimated affinity of − 5.1 kcal/mol. Further, PAT bonded with ADRA1 though interactions with non-thiol containing amino acids side chains of serine (ser), tyrosine (tyr), phenylalanine (phe), glutamic acid (glu) and lysine (lys) (Fig. 5e), indicative of a novel binding. Interestingly, Epi was predicted to bind at a different location. It was determined that Epi had significant interactions with the side chains of glycine (gly), leucine (leu), aspartic acid (asp), proline (pro), valine (val), phenylalanine (phe) and glutamic acid (glu) (Fig. 5f). The binding affinity of Epi to ADRA1 was stronger than PAT with an estimated affinity of − 6.5 kcal/mol (Fig. 5g,h).

### PAT alters α_1_-AR associated ERK/MAPK signalling in HEK293 cells

**Figure 6:**
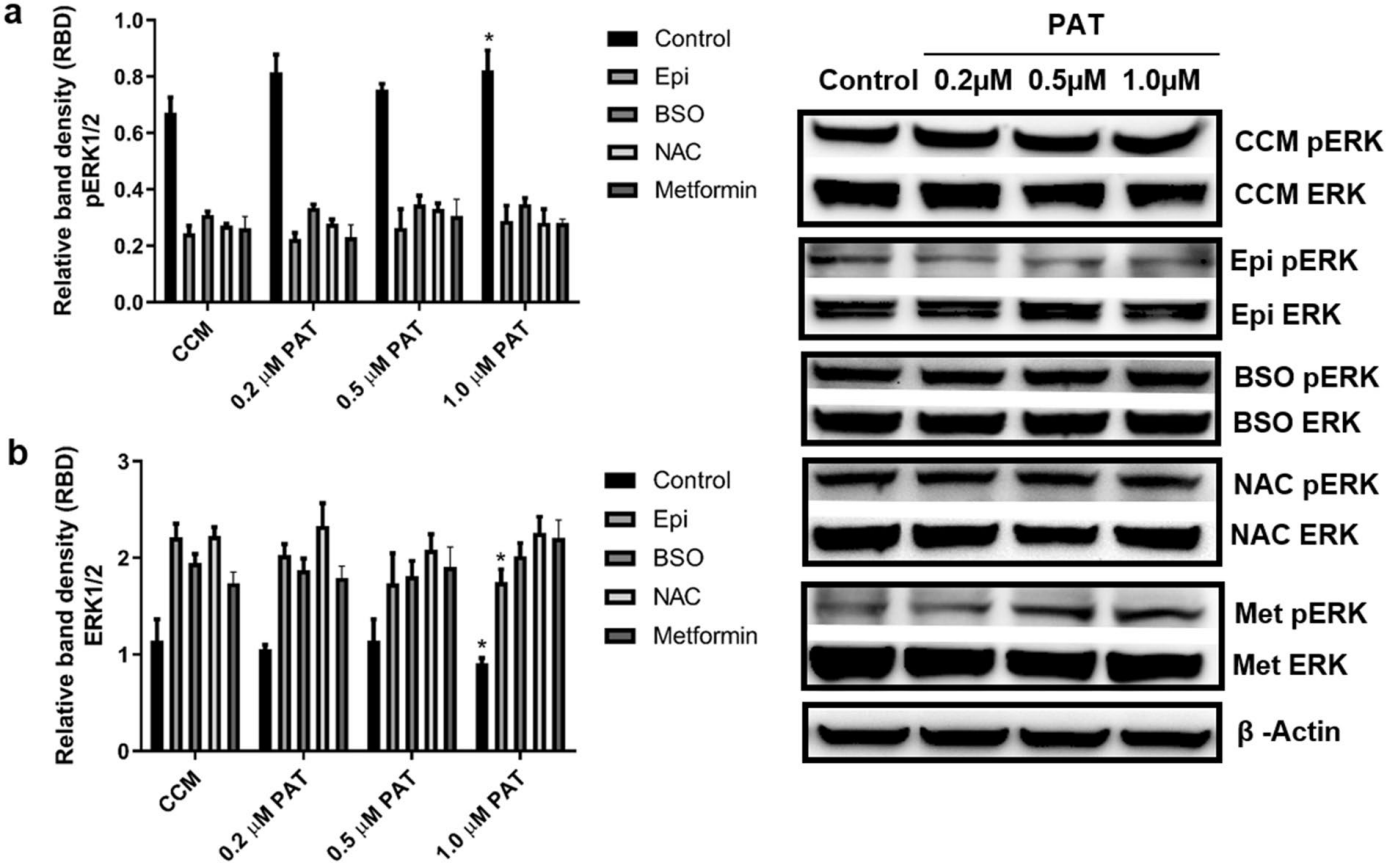
PAT alters α_1_-AR associated ERK/MAPK signalling. Western blotting established PAT significantly increased ERK1/2 phosphorylation (**a**) activation relative to total ERK expression, (**b**) in HEK293 cells following 24 h exposure. A comparable trend was observed in Epi-simulated cells following PAT administration, while metformin, BSO and NAC pre-treatments had no significant effects on the pathway following PAT exposure (*p < 0.05 relative to respective control). The original western blots are included in Supplementary Data S8.

ERK1/2 activation was measured to determine whether changes in α_1_-AR expression affected downstream signalling. In the controls PAT significantly decreased expression of total ERK1/2 (p = 0.0429; Fig. 6b) and concomitantly increased pERK1/2 (p = 0.0269; Fig. 6a), most notably at 1 µM. Similarly, in cells pre-exposed to Epi, total ERK1/2 expression (p = 0.0332; Fig. 6b) was significantly decreased with corresponding increases in pERK1/2 expression (p = 0.0471; Fig. 6a) and prominent changes in 1 µM treatments. This was indicative of increased pERK1/2 activation under both conditions. Investigations into the role of thiol depletion determined BSO had no effect on expression of total ERK1/2 (p = 0.3611; Fig. 6b) and pERK1/2 (p = 0.1267; Fig. 6a). NAC treatments also had no effect on total ERK1/2 (p = 0.3916; Fig. 6b) and pERK1/2 expression (p = 0.1916; Fig. 6a). Similarly, there was no observed change in pERK1/2 (p = 0.2606; Fig. 6a) and ERK1/2 (p = 0.1112; Fig. 6b) activation in metformin treatments.

### PAT alters PI3K/Akt signalling in HEK293 cells

**Figure 7:**
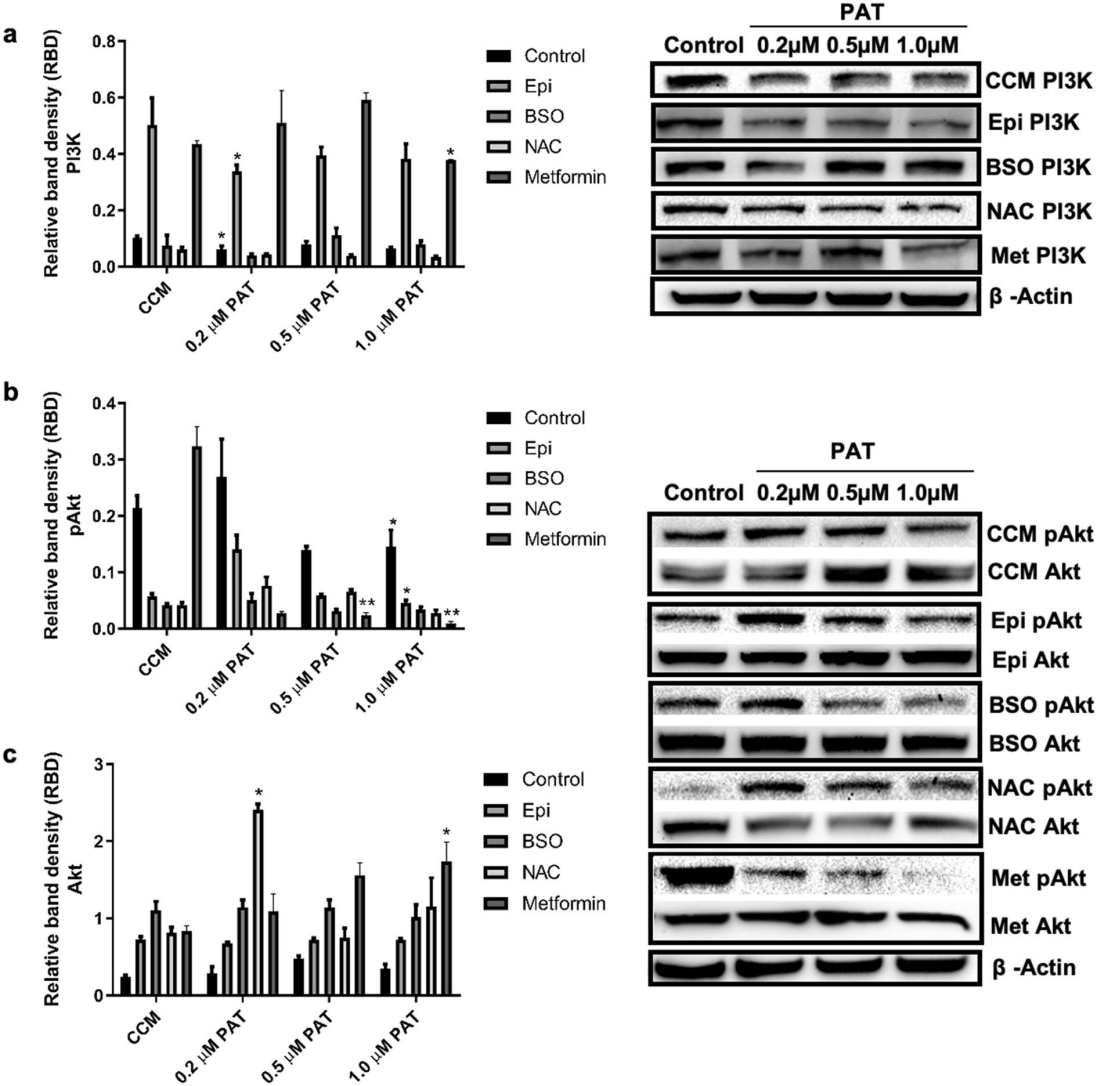
PAT alters PI3K/Akt signalling in HEK293 cells. Western blotting revealed PAT significantly deceased PI3K/Akt signalling. Similar trends were observed in Epi-stimulated and NAC treated cells, while cells pre-exposed to BSO showed no statistically significant changes. Metformin treatments however showed a significant in decrease in pAkt following PAT exposure (*p < 0.05 relative to respective control). The original western blots are included in Supplementary Data S9 and S10.

Downstream PI3K (p110γ) expression and Akt signalling were investigated using western blotting. A significant decrease in PI3K (p = 0.0103, Fig. 7a) with commensurate changes in pAkt (p = 0.0218, Fig. 7b) and Akt (p = 0.0415, Fig. 7c) expression was observed following PAT exposure. A similar trend was observed in cells pre-exposed to α_1_-AR agonist, Epi. A significant decrease in PI3K (p = 0.0378, Fig. 7a) with an associated decrease in pAkt activation (p = 0.0232, Fig. 7b) was shown with no change in total Akt (p = 0.1916, Fig. 7c). GSH depletion (represented by BSO treatments) induced no significant changes to the pathway as evidenced by unchanged PI3K (p = 0.586, Fig. 7a), pAkt (p = 0.286, Fig. 7b) and total Akt (p = 0.5777, Fig. 7c) expression. NAC treated cells however significantly decreased PI3K expression (p = 0.0232, Fig. 7a) with a dose dependent decrease in pAkt (p = 0.0237, Fig. 7b) and an associated dose dependent increase in total Akt (p = 0.0378, Fig. 7c). Metformin treatments, representative of AMPK activation indicated PI3K expression was significantly decreased by 1 µM PAT (p = 0.0348). Despite significant phosphorylation and activation of Akt by metformin, PAT decreased pAkt in the treatments (p = 0.001) and concomitantly increased Akt expression (p = 0.006) in these treatments. These results indicate that the effects of PAT on the PI3K/Akt pathway may involve an alternate means of regulation beyond GSH depletion.

## Discussion

The α_1_-AR mediates the effects of catecholamines throughout the human body^36^. These receptors are functionally linked to renal tone, metabolism, tubule function and sodium (Na^+^) reabsorption in the kidney—where persistent suppression of α_1_-AR is associated with renal fibrosis^15,16,19,21,37,38^. This study showed that PAT altered the transcription and translation of α_1_-AR in HEK293 cells (Figs. 2, 3, 4). This finding was consistent across toxic, safety, environmentally and physiologically relevant concentrations, presenting insight into PAT-induced renal toxicity (Figs. 2, 3, 4).

PAT, often found in mould-contaminated apples is a strongly electrophilic molecule and exerts toxicity by binding thiol groups. GSH is a prominent cellular thiol-containing compound involved in redox homeostasis. PAT has been linked extensively to GSH depletion and oxidative stress in the kidney^8,9,35^. PAT-induced suppression of α_1_-AR was potentiated in the presence of GSH inhibitor, BSO (Fig. 4b). GSH supplementation with NAC ameliorated this effect, alluding to thiol group depletion as a possible mechanism (Fig. 4c). Interestingly, molecular modelling challenged this finding (Fig. 5). These results showed PAT interacted directly with ADRA1 though hydrogen bonding, pi bonding and alkyl bonding with the side chains of Ser, Tyr, Phe, Glu and Lys (Fig. 5). None of the amino acids indicated contain a thiol side chain, revealing a novel mode of PAT affinity and binding. This finding offers an additional explanation for the low ADRA1 protein expression in PAT treatments (Figs. 2, 3, 4) and offers new insights into PAT induced toxicity beyond GSH inhibition and ROS generation. Most notably, PAT opposed the actions of α_1_-AR pathway agonist Epi and AMPK activator, metformin. A significant decrease in α_1_-AR was observed following PAT exposure in Epi (Fig. 4a) and metformin (Fig. 4d) pre-treatments.

The ADRA1 protein results however, should be interpreted with caution. The amino acid sequence corresponding to the epitope for the α_1_-AR antibody used in this study is conserved between all three ADRA1 receptor subtypes—presenting a limitation due to potentially low specificity. In addition, overall protein expression may not correspond directly with plasma membrane localization or mRNA levels. Together, this may account for the increase in α_1_ receptor subtype-specific mRNA levels in some PAT treatments (Fig. 3) despite consistently observed decreases in overall ADRA1 protein expression. As such, while the PAT molecular docking studies support overall protein findings, the radio ligand binding assay, generation of crystal structures and/or NMR is required to conclusively prove a mechanism of inhibition, (e.g., antagonism, reverse agonism or shift from active to inactive state)—presenting an interesting avenue for future studies^39–43^. In addition, potential PAT-induced changes to transcriptional, translational and post-translational processes pertaining to α_1_-AR expression could be explored. Nevertheless, the findings in this study suggest a novel role for PAT in AMPK and Epi-mediated signalling and metabolism with previously unknown molecular targets. This finding may be mechanistically linked to altered renal and cellular function discussed in previous studies on PAT^34,44–50^.

PAT exposure has been associated with impaired kidney function characterised by degeneration of glomeruli, haemorrhage in the tubules and cortical regions, tubular atrophy and diminished clearance abilities—hallmarks of kidney injury and disease^10,49–51^. AMPK has a unique regulatory role in the kidney at the junction of energy metabolism, ion transport, inflammation and stress. Molecular docking studies indicated PAT interacted directly with Arg and Hie residues on PRKAG3 through hydrogen bonding (Fig. 1). This was supported by findings in the Human AMPK Signalling PCR Array (Supplementary Data S3) and western blotting which showed PAT exposure opposed effects of AMPK activator metformin on ADRA1 protein expression (Fig. 3). α_1_-AR mediated AMPK activation; suppressed in the early stages of kidney injury and disease^16^—was also suppressed following PAT exposure in this study (Figs. 2, 3, 4)—supporting a novel mechanism for PAT-induced kidney injury. This is associated with reduced tubule function, decreased kidney mass and fibrosis. Hence this pathway has a potential role to play in modulation of PAT-induced kidney injury and progression of acute and chronic kidney disease^18^.

Studies on α_1_-AR ligand-binding report that occupancy of the receptor can trigger direct or parallel activation of—MAPK/ERK1/2. This provides evidence that α_1_-AR can activate ERK with or without the canonical GPCR pathway^42^. This is an important consideration given the different predictive modeling binding sites of PAT and Epi (Figs. 5, 6). The MAPKs play a central role in cellular signal transduction between the cell surface and nucleus. This study showed ERK1/2 signalling was increased following PAT exposure (Fig. 6), a finding corroborated by literature^1^. This trend was consistent following Epi stimulation and neutralised by metformin and NAC, no changes were noted in BSO treatments however, which may be related to direct effects of BSO on this redox sensitive pathway (Fig. 5)^52^. While the observed Epi trend was consistent with other findings, the ERK1/2 pathway integrates diverse signalling pathways and stimuli including growth factors, tyrosine kinase or GPCR mediated activation. Epi effects on the ERK1/2 pathway are also mediated through α_2_- and β- adrenergic receptors, which are potentially confounding factors in relation to this study parameter^15^. Future studies using receptor blockers are required to fully understand the effects observed on the pathway. With respect to α_1_-AR signalling—PKC and Ca^2+^ can activate the ERK pathway directly which phosphorylates and activates other protein kinases and transcription factors involved in survival, proliferation and apoptosis—all of which have been linked to PAT-toxicity^53–55^. An investigation into potential changes in Ca^2+^ flux resulting from PAT exposure could reveal further connections between these signaling networks and associated toxic outcomes.

While the ERK1/2/MAPK pathway has been linked directly to α_1_-AR signalling; debate surrounds the distinct activation and function of α_1_-AR activated PI3K signalling^20,25,27,28,56^. This study showed PAT suppressed PI3K/Akt signalling, even when stimulated with Epi and metformin (Fig. 6). α_1_-AR stimulated PI3K activation was also shown in murine keratinocytes^56^. Evidence suggests this has a functional role in glycogen regulation and receptor desensitization; which may be supported by findings in this study (Supplementary Data S3) and others showing PAT alters PI3K/Akt signalling in the same cells^20,28,31^. Several other studies however have found this pathway is primarily activated via co-ordinated cross talk between ERK1/2/MAPK pathways, Epi-stimulated signalling and receptor tyrosine kinases (RTK)^20,27,57^; providing further substantiation for PAT-induced suppression of the pathway and Epi-mediated action (Fig. 6). These pathways have been linked to DNA synthesis, cell cycle progression, proliferation and metabolic regulation—often associated with carcinogenesis. Our findings indicated PAT slowed the kidney metabolic machinery and proliferation pathways—as a possible energy conservation strategy, evidenced by decreased PI3K/Akt activation (Fig. 7). The potential role for the mitochondria and AMPK in PAT-mediated toxicity is supported by significantly reduced PI3K/Akt activation in PAT-exposed metformin pre-treatments (Fig. 7). Suppression of this pathway is also linked to apoptosis and cell death via mitochondrial dysfunction—a key feature of PAT toxicity^29,30^. This is shown in literature confirming PAT impairs ATP and mitochondrial function, causes DNA damage, cell cycle arrest and cell death^11,34,53,55,58,59^.

Stress response mechanisms determining cell death and survival are central modulators in recovery and progression of renal injury. The apoptotic response to stress in the kidney; closely associated with PAT exposure in vitro and in vivo—is a critical event in the loss of tubular epithelial cells observed in kidney injury. AMPK signal transduction via α_1_-AR mediates cell survival, repair and mitochondrial biogenesis. This study shows the pathway was suppressed by PAT-directly and through thiol depletion—providing a mechanistic explanation for previous cell death, mitochondrial impairment and nephrotoxicity studies on PAT^1,32,34,48,49^.

Literature indicates PAT is nephrotoxic and has been associated with oxidative stress, cell cycle changes, apoptosis, diminished clearance abilities and changes in blood flow^34,44–46,60^. This is the first study to show PAT alters α_1_-AR signalling and Epi-mediated action on the pathway, associated with changes in downstream signalling. This provides renewed insight to previous physiological and descriptive data on PAT-mediated toxicity. The distribution of these receptors in the brain, heart, vasculature and liver together with the functional relevance of Epi-mediated signalling and PAT target organs, warrants further investigation into PAT-induced changes in this pathway.

In conclusion, PAT-induced decreases in transcription and translation of α_1_-AR was associated with changes in downstream PI3K and MAPK signalling—most significantly at 1 µM, above safety level concentrations, supporting existing regulation and the need for food monitoring. This effect was consistent in cells stimulated with pathway agonist Epi and AMPK activator metformin—suggestive of a suppressive role for PAT in Epi- and AMPK-mediated signalling. The effects of this parameter on downstream signalling was inconclusive and requires further investigation. Future studies including both pre-treatments and post-treatments, radio ligand binding assays, α_1_-AR and β-AR antagonists could elucidate mechanistic data and improve understanding of the downstream signalling observed. This study provides significant insights to previous studies on PAT and yields potential for further investigation.

## Materials and methods

### Materials

HEK293 cells were purchased from Highveld Biologicals (Johannesburg, South Africa). Culture reagents were purchased from Lonza Bio-Whittaker (Basel, Switzerland). Patulin (P1639) was purchased from Sigma-Aldrich (St Louis, USA). Western Blotting and RT-PCR reagents and consumables were obtained from Bio-Rad (Hercules, USA) and all other reagents were received from Merck (Darmstadt, Germany) unless otherwise stated.

### Cell culture

HEK293 cells were cultured in complete culture medium (CCM) comprising Dulbecco’s minimum essential medium (DMEM) supplemented with 2 mM l-glutamine, 1% penstrepfungizone (500 units potassium penicillin, 500 μg streptomycin/5 ml flask), 10 mM HEPES, 10% fetal bovine serum in 25 cm^3^ flasks at 37 °C; 5% CO_2_. When cells were 90% confluent, cells were treated with PAT (0.2 µM; 0.5 µM; 1 µM; 2.5 µM) for 24 h (h). PAT (5 mg) was dissolved in 1 ml 100% dimethyl sulfoxide (DMSO). PAT stock solutions were prepared in 0.1 M PBS to a final concentration of 1 mM. Control experiments were represented by the maximum amount of DMSO in CCM (0.00018%).

### Dosage information

#### Rationalization

The most commonly cited PAT incidence in consumables ranges between 0.1 and 4 μM despite the 50 μg/l (0.3 μM) safety level regulation^3,61–63^.

A study by Dailey et al. in both male and female rats showed 36% PAT was recovered in the urine 7 days post-administration^64^. This study found PAT accumulated in blood rich organs specifically the kidney, liver, erythrocytes and spleen. While current data on the absorption, distribution, metabolism, and excretion of PAT are limited, other studies have confirmed the bioaccumulation of PAT in the kidney. An in vivo study in albino mice found 1 μM oral administration of PAT led to glomerular haemorrhage, damage to the cortical regions and tubules of the kidney^48^. Another study (in 2012) using 6-22 μM PAT in mice also reported kidney damage and established a link between these effects and GSH depletion^50^. A recent in vitro study on HEK293 (kidney) cells by Jin and colleagues (2016) selected a range of 0–9 μM PAT over 24 h to determine a role for p53 in PAT mediated kidney damage^3,48,59^. The highest concentration used in our study (2.5 μM) is below the upper range cited in previous PAT exposure investigations—but within range of concentrations reported in incidence studies. Previously, a range of PAT concentrations (0–100 μM) was tested on HEK293 cell viability (using the MTT assay) following 24 h exposure^33^. The MTT assay indicated 100 µM PAT reduced HEK293 cell viability to 2% following 24 h exposure and an IC_50_ of 2.5 μM was determined.

In the current study 2.5 μM (previous IC_50_) represents the highest concentration of PAT used. Interestingly, this concentration corresponds to approximately 36% less than the upper concentration of 4 μM PAT (from literature)—accounting for clearance and recovery; while the lowest concentration used was 0.2 μM—below the established safety level (0.3 μM), accounting for lower range incidences.

Hence, the concentration range selected in our study (0.2 μM; 0.5 μM; 1 μM; 2.5 μM), in general, relates to the incidence of PAT found in food and beverages and accounts for clearance, recovery and retention levels as reported in previous studies.

#### Treatment conditions

Preliminary experiments used toxic exposure (2.5 µM PAT) determined from cytotoxic assays in previous studies using the same model^33,34^. Concentrations relevant to environmental and safety levels (0.2 µM; 0.5 µM; 1 µM PAT)—determined from incidence, monitor studies and literature were used to validate findings and molecular mechanisms in subsequent assays^2,3^.

Experiments examining the effects of PAT on ADRA1 protein signalling were exposed to PAT as described above. Cells were preincubated with α_1_-AR agonist Epi (Sigma, St Louis, USA) 10 µM for 30 min (min) prior to PAT exposure and further incubated for 24 h^36^. Buthionine Sulphoximine (BSO) 5 mM (Sigma, St Louis, USA) and N-acetylcysteine (NAC) 2 mM were preincubated for 1 h followed by the PAT exposure, to simulate GSH depletion and supplementation, respectively (Supplementary Data S1). This was included to determine whether observed effects were related to previously established mechanisms of PAT toxicity^65^. Experiments conducted using cells pre-exposed to AMPK activator metformin (5 mM) for 30 min—5 mM concentration was selected from literature^66^.

### Quantitative PCR

#### cDNA preparation

Total RNA was isolated using QIAzol lysis reagent (Qiagen, Hilden, Germany). Briefly, QIAzol (500 µl) was added to 1 × 10^6^ cells in 500 µl 0.1 M PBS and incubated overnight (− 80 °C). Chloroform (100 µl) was added and samples were then centrifuged (15 min, 12,000×*g*, 4 °C). The aqueous phase was added to 250 µl isopropanol in a clean tube and incubated overnight (− 80 °C). Samples were centrifuged (20 min, 12,000×*g*, 4 °C) and supernatant was discarded. Pellets were washed with 500 µl cold ethanol and centrifuged (15 min, 7400×*g*, 4 °C). Ethanol was removed; samples were dried and resuspended in 12.5 µl RNase-free water. RNA was quantified using the Nanodrop2000 and standardized to 1000 µg/μl.

#### Determination of differentially regulated genes

Quantitative PCR (qPCR) was used to identify the relative expression of 84 genes involved in AMPK signalling. cDNA was prepared using the RT2 cDNA synthesis kit (330401) (Qiagen, Hilden, Germany) and RT2 PreAMP Pathway Primer Mix for the Human AMPK Signalling Array (PBH-175Z) (Qiagen, Hilden, Germany) according to the manufacturer’s instructions. The RT^2^ Profiler Human AMPK Signalling PCR Array (PAHS-175Z) (Qiagen, Hilden, Germany) was used. cDNA template of control and PAT samples was added to the mastermix according to the manufacturer’s instructions. The CFX Touch Real Time PCR Detection System (Bio-Rad) was used. The reaction was subjected to initial denaturation (95 °C, 10 min), followed by 40 cycles (95 °C, 15 s), a plate read (60 °C, 1 min). Five internal housekeeping genes and quality control were included in the array. Relative fold change was determined using the online Data Analysis Center by Qiagen. Fold changes of ≥ 2 in PAT arrays differentially regulated compared to the control were considered significant.

#### Array validations

Differentially regulated genes: *ADRA1A, ADRA1D, ADRA2A, ADRA2B* were validated using qPCR. cDNA was synthesised using iScript cDNA Synthesis kit (Bio-Rad; catalogue no 107-8890) as per manufacturer’s instructions. Thermocycler conditions were 25 °C for 5 min, 42 °C for 30 min, 85 °C for 5 min and a final hold at 4 °C. mRNA levels were evaluated using the SsoAdvanced Universal SYBR Green Supermix (Bio-Rad; 170-880) according to the manufacturer’s instructions. cDNA template was added to 30 nM sense primer, 30 nM antisense primer, reaction mix and nuclease free water was made up to a reaction volume of 25 µl. Primer sequences and annealing temperatures are as follows *ADRA1A* [Sense 5′-ATGCTCCAGCCAAGAGTTCA-3′; Antisense 5′-TCCAAGAAGAGCTGGCCTTC-3′ (60 °C)]; *ADRA1D* [Sense 5′-TTATGGCCGTGGCAGGTAAC-3′; Antisense 5′-GCCAGGTTCACGATGAAATAGTT-3′ (60 °C)]; *ADRA2A* [Sense 5′-AAACCTCTTCCTGGTGTCTC-3′; Antisense 5′-AGACGAGCTCTCCTCCAGGT-3′ (58 °C)]; *ADRA2B* [Sense 5′-CCTGGCCTCCAGCATCGGAT-3′; Antisense 5′-ATGACCACAGCCAGCACGAA-3′ (58 °C)]. All assays were carried out using the CFX Touch Real Time PCR Detection System (Bio-Rad). The reaction was subjected to initial denaturation (95 °C, 4 min), followed by 37 denaturation cycles (95 °C, 15), annealing (primer-specific temperature, 40 s), extension (72 °C, 30) and a plate read for 37 cycles. *β actin* [Sense 5′-TGACGGGTCACCCACACTGTGCCCAT-3′; Antisense 5′-CTAGAAGCATTTGCGGTGGACGATGGAGGG-3] and *GAPDH* [Sense 5′-TCCACCACCCTGTTGCTGTA-3′; Antisense 5′-ACCACAGTCCATGCCATCAC-3′] were run under the same conditions and used as internal housekeeping genes. Data was analysed using methodology described by Livak and Schmittgen^67^ and is represented as fold change (2^−ΔΔCT^) relative to the housekeeping genes.

### Western blotting

#### Protein isolation

Cytobuster (Novagen, South Africa) supplemented with protease inhibitor and phosphatase inhibitor (Roche 05892791001 and 04906837001, respectively) was added to cells (4 °C, 10 min) and centrifuged (5 min, 10,000×*g*, 4 °C). The supernatant representing crude protein extracts was quantified using the bichinchonic assay (Sigma, St Louis, USA) and standardized to 1 mg/ml. Samples were then denatured by boiling (5 min, 100 °C) in Laemmli buffer (dH_2_O, 0.5 M TrisHCl (pH 6.8), glycerol, 10% sodium dodecyl sulphide polyacrylamide (SDS), β-mercaptoethanol, 1% bromophenol blue).

#### Sodium dodecyl sulphate–polyacrylamide gel electrophoresis (SDS-PAGE) and immunoblotting

Samples were electrophoresed on 7% SDS gels (1 h, 150 V) and transferred to nitrocellulose membranes using the TransBlot Turbo System (Bio-Rad). Membranes were blocked (1 h, 3%, bovine serum albumin (BSA)) in Tris buffered saline with Tween20 (TTBS) (20 mM Tris–HCl; pH 7.4), 500 mM NaCl and 0.01% Tween 20). Membranes were then incubated with primary antibody (ADRA1 (ab3462); p44/42 MAPK (ERK1/2) (CST#9102); phosphor-p44/42 MAPK (ERK1/2) (Thr202/Try204) (CST#9106); PI3K p110γ (CST#5405); phosphor-ser473 Akt (CST#9271); Akt (CST#9272); (1:1000) in 1% BSA in TTBS overnight at 4 °C. Membranes were washed five times (10 ml, TTBS, 10 min) and treated with horseradish peroxidase-conjugated secondary antibody (anti-rabbit, CST #7074; anti-mouse, CST #7076, 1:10,000) in 1% BSA (1 h, RT). Membranes were then washed five times (10 ml, TTBS, 10 min) and immunoreactivity was detected (Clarity Western ECL Blotting Substrate, Bio-Rad) with the Bio-Rad Chemidoc Imaging System. Protein bands were analyzed with the Bio-Rad Image Lab Analysis 6.0 software and normalized against the corresponding β Actin bands.

#### Molecular docking

Molecular docking was performed to determine a mechanism of substrate/inhibitor selectivity and to visualize the ligand orientation and the active site cavity of the protein^68^. The protein sequence of human ADRA1A and PRKAG3 was retrieved from PubMed. The 3D model of ADRA1 and PRKAG3 was generated using Homology modeling and Modeler software^69^. The chemical structure of PAT was drawn using Chem draw software. Molecular docking is a technique to predict the preferential orientation between two molecules to form a stable complex. Docking calculations are done to investigate the binding affinity between selected small molecules and target proteins. Glide docking requires a receptor grid and a set of ligand structures for flexible docking using the Monte Carlo based simulated algorithm. This technique was carried out using Glide v12.1 in which Glide SP and XP were applied. SP docking was done to screen ligands, which were large in number, and then XP docking was done which is more powerful as its run time is longer than SP. XP docking uses Extra precision and write XP descriptor information generates favorable ligand poses which were further screened through filters to examine the spatial fit of the ligands in the active site. Ligand poses which pass through initial screening are subjected to the evaluation and minimization of grid approximation. In the grid-based docking technique, the receptor is basically rigid. XP mode is tolerant than SP mode because it can screen out the false positive. XP is designed to place the active ligands that bind to the receptor in particular conformation. The best pose of each ligand was ranked based on Glide XP Glide score^70^.

### Statistical analysis

Results are represented as mean ± standard deviation (SD) relative to normalized control. This is representative of three independent experiments completed in triplicate and error bars showing standard deviation. Statistical significance was assessed using t tests, one-way and two-way ANOVA with appropriate post hoc comparisons on GraphPad Version 5.0 Software. p values less than 0.05 were considered significant.

## Supplementary information

Supplementary Information.
