# Supplementary material for: Patulin suppresses α1-adrenergic receptor expression in HEK293 cells
Source: Sci Rep. 2020 Nov 18;10:20115. doi: 10.1038/s41598-020-77157-0 (PMC7674415; doi:10.1038/s41598-020-77157-0)
Supplement: Supplementary file 1 — Supplementary Information. [file 41598_2020_77157_MOESM1_ESM.docx]

**Patulin suppresses α_1_-adrenergic receptor expression in HEK293 cells**

Yashodani Pillay, Savania Nagiah, Alisa Phulukdaree, Anand Krishnan, Anil A. Chuturgoon

Discipline of Medical Biochemistry, University of KwaZulu-Natal, Durban, South Africa

**Supporting Information:**

**Materials and Methods:**

Glutathione Assay (Supplementary data S1)

The GSH-Glo Glutathione Assay (Promega, Madison, USA) was used to measure reduced GSH levels. PAT, BSO and NAC treated cells were transferred to an opaque microtiter plate (50µL of 20,000 cells/well; 6 replicates). GSH standards (0 – 5µM) were prepared from a 5mM stock solution diluted in PBS. Five two-fold dilutions of the GSH stock were prepared and transferred into wells (50µL) of the microtiter plate. The 2X GSH-Glo Reagents were prepared according to the manufacturer’s instructions, added to the experimental wells (50µL/well), and incubated at room temperature (RT, 30min). Reconstituted Luciferin Detection Reagent (50µL) was added to each well and incubated (RT, 15min) before the luminescence was measured (Modulus microplate luminometer, Turner Biosystems, Sunnyvale, USA). A standard curve was derived and the GSH concentration in each sample was extrapolated from the equation.

ATP Assay (Supplementary data S2)

Cells (20,000/well in six replicates) were aliquoted in an opaque 96-well microtiter plate to which the ATP CellTiter Glo (Promega, Madison, USA) reagent (50μl) was added and allowed to react in the dark (RT, 30min). After incubation, the luminescent signal proportional to the cellular ATP content was detected with a Modulus microplate reader (Turner Biosystems, Sunnyvale, USA). The results were expressed graphically as mean relative light units (RLU) ± standard deviation.

JC-1 MitoScreen Assay (Supplementary data S2)

Mitochondrial membrane potential (Δψm) was assayed using the JC-1 BD MitoScreen kit (BD Biosciences). JC-1 working solution was prepared and 100μl added to each tube, followed by 100μl of cell suspension. Tubes were incubated at 37°C with 5% CO_2_ for 15min, after which 100μl of JC-1 wash buffer was added. Approximately 50,000 events were analyzed on an Accuri C6 flow cytometer. Events were gated and analyzed using CFlow Plus Software. The results were expressed as percentage.

Mitochondrial DNA (mtDNA) Viability (Supplementary data S2)

DNA was extracted from PAT treated cells using the method defined by Sambrook et al., 2001 [2]. The DNA was quantified (Nanodrop 2000) and standardised (10ng/µl). A 25µl reaction volume containing 4μl DNA template, 10ρmol sense primer, 10ρmol antisense primer, 5X iScript reaction mix and nuclease free water was used. All assays were carried out using CFX Touch Real Time PCR Detection System (Bio-Rad). The reaction was subjected to initial denaturation (95˚C for 3min), followed by 28 denaturation cycles (95˚C, 20sec), annealing (58˚C, 10min), extension (72˚C, 10min) followed by a plate read for 37 cycles. β-Globin was run under the same conditions and used as the housekeeping gene. Primer sequences were as follows: mtDNA- Forward 5’-TGAGGCCAAATATCATTCTGAGGGC-3’; mtDNA- Reverse 5’-TGCACCTGCTCTCTGTGATTATGACTATCCCACAGTC-3’; β-Globin- Forward 5’-ACATGATTAGCAAAGGGCCTAGCTTGACTCAGA-3’; β-Globin- Reverse 5’-TGCACCTGCTCTGTGATTATGACTATCCCACAGTC-3’. Results are represented as mean fold change ± SD.

Cell culture (Supplementary data S3)

Metformin, an AMPK activator was used to assess the effects PAT on the AMPK pathway. Cells were preincubated with 5mM metformin for 30 minutes as determined using literature (He and Wondisford 2015) for maximal activation before addition of PAT. Protein was isolated and western blotting was completed according to the protocol previously described in the manuscript. Results are represented as relative band density ± SD.

**Supplementary Data S1:**

PAT depletes GSH in HEK293 cells

The Luminometric GSH-Glo Glutathione Assay was used to verify previous GSH findings on PAT and the use of 5mM BSO and 2mM NAC as positive and negative controls in this study. PAT depleted GSH 1.3-fold in HEK293 cells – confirming previous findings. The selected BSO concentration followed a similar trend, causing a highly significant 4-fold depletion in GSH while NAC increased GSH 1.5-fold – directly opposing the effect of PAT.


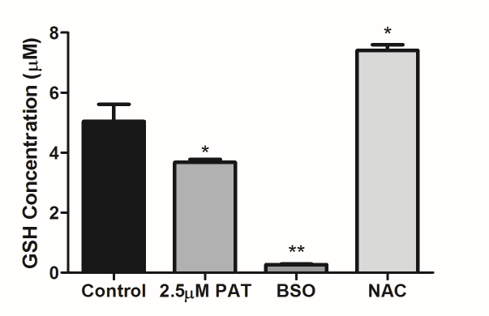


**Fig.A** PAT depleted GSH. Luminometric analysis indicated 2.5µM PAT, 5mM BSO and 2mM NAC significantly alters GSH concentrations following 24h exposure in HEK293 cells p=0.0027 *(**p<0.01;* **p<0.05 relative to control)*

**Supplementary Data S2**

PAT compromises ATP production via diminished mitochondrial function

Mitochondrial transcription factor A (TFAM) is an essential regulator of mitochondrial DNA transcription and copy number. qPCR determined 2.5µM PAT decreased TFAM levels 1.6-fold (p=0.0125; Fig7A). This was accompanied by a significant 7-fold decrease in mitochondrial DNA (mtDNA) integrity as determined with the mtDNA assay (p=0.0294; Fig7B). Flow cytometry JC-1 stain indicated PAT increased mitochondrial depolarization by 15 % (p=0.0050; Fig 7C) and concomitantly decreased ATP levels 1.18-fold (p=0.0294; Fig7D) as assessed using the ATP CellTiter Glo Assay. The compromised mitochondrial integrity and function observed provided the rationale for examination of pathways mediated by energetics regulator AMPK.


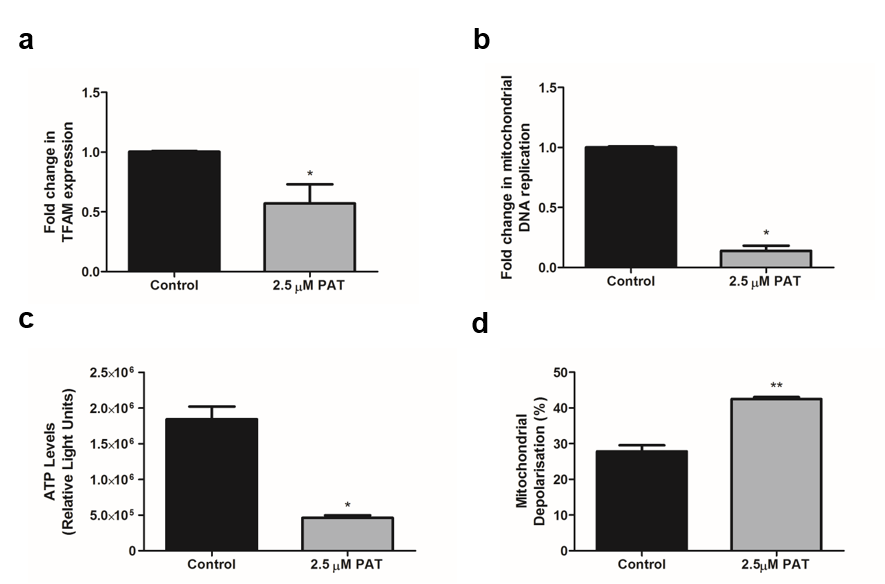


**Fig.B** PAT compromises ATP production via diminished mitochondrial function. A) qPCR results determined decreased TFAM expression (p=0.0125) B) qPCR results for mtDNA amplification were indicative of vastly impaired mtDNA integrity (p=0.0294) C) JC-1 stain indicated increased mitochondrial depolarization in PAT treated cells (p=0.0050) corresponding with D) ATP Assay results showing a decrease in ATP (p=0.0294) *(**p<0.01;* **p<0.05 relative to control)*

**Supplementary Data S3**

Table A. Human AMPK Signalling PCR Array results showing differentially regulated gene expression

| Group | Gene Symbol | Description | Fold Change |
| --- | --- | --- | --- |
| Alpha Adrenergic Receptors |  |  |  |
|  | *ADRA1A* | Adrenergic, alpha 1A-, receptor | -2.264* |
|  | *ADRA1B* | Adrenergic, alpha 1B-, receptor | -1.7113 |
|  | *ADRA1D* | Adrenergic, alpha 1D-, receptor | -2.5168* |
|  | *ADRA2A* | Adrenergic, alpha 2A-, receptor | -2.6626* |
|  | *ADRA2B* | Adrenergic, alpha 2B-, receptor | -3.9068* |
|  | *ADRA2C* | Adrenergic, alpha 2C-, receptor | -1.5858 |
| Hormone Receptors |  |  |  |
|  | *ADIPOR1* | Adiponectin receptor 1 | 1.0382 |
|  | *ADIPOR2* | Adiponectin receptor 2 | 1.0600 |
|  | *INSR* | Insulin receptor | -1.1879 |
|  | *LEPR* | Leptin receptor | -1.4756 |
| Nicotinic Receptors |  |  |  |
|  | *CHRNA1* | Cholinergic receptor, nicotinic, alpha 1 | -3.157* |
|  | *CHRNB1* | Cholinergic receptor, nicotinic, beta 1 | -1.2985 |
| Adenylate Kinases |  |  |  |
|  | *AK1* | Adenylate kinase 1 | -1.2409 |
|  | *AK2* | Adenylate kinase 2 | -1.3846 |
|  | *AK3* | Adenylate kinase 3 | -1.0780 |
| Akt and PI3K Signalling |  |  |  |
|  | *AKT1* | V-akt murine thymoma viral oncogene homolog 1 | -1.0619 |
|  | *AKT2* | V-akt murine thymoma viral oncogene homolog 2 | -1.0584 |
|  | *AKT3* | V-akt murine thymoma viral oncogene homolog 3 | -1.0931 |
|  | *PDPK1* | 3-phosphoinositide dependent protein kinase | 1.0303 |
| Calcium/Calmodulin Signalling |  |  |  |
|  | *CAMKK1* | Calcium/calmodulin protein kinase kinase 1, alpha | 1.0624 |
|  | *CAMKK2* | Calcium/calmodulin protein kinase kinase 2, beta | -1.1637 |
| AMPK Catalytic Subunits |  |  |  |
|  | *PRKAA1* | Protein kinase, AMP-activated, alpha 1 catalytic subunit | -1.2182 |
|  | *PRKAA2* | Protein kinase, AMP-activated, alpha 2 catalytic subunit | -1.1684 |
| AMPK Regulatory Subunits |  |  |  |
|  | *PRKAB1* | Protein kinase, AMP-activated, beta 1 non-catalytic subunit | 1.0353 |
|  | *PRKAB2* | Protein kinase, AMP-activated, beta 2 non-catalytic subunit | -1.1269 |
|  | *PRKAG1* | Protein kinase, AMP-activated, gamma 1 non-catalytic subunit | -1.0201 |
|  | *PRKAG2* | Protein kinase, AMP-activated, gamma 2 non-catalytic subunit | 1.0345 |
|  | *PRKAG3* | Protein kinase, AMP-activated, gamma 3 non-catalytic subunit | -4.1898* |
| Protein Kinase A Catalytic Subunits |  |  |  |
|  | *PRKACA* | Protein kinase, cAMP-dependent, catalytic alpha | 1.0701 |
|  | *PRKACB* | Protein kinase, cAMP-dependent, catalytic beta | -1.0187 |
| Protein Kinase A Regulatory Subunits |  |  |  |
|  | *PRKAR1A* | Protein kinase, cAMP-dependent, regulatory, type I, alpha | 1.1052 |
|  | *PRKAR1B* | Protein kinase, cAMP-dependent, regulatory, type I, beta | 1.1621 |
|  | *PRKAR2A* | Protein kinase, cAMP-dependent, regulatory, type II, alpha | -1.1108 |
|  | *PRKAR2B* | Protein kinase, cAMP-dependent, regulatory, type II, beta | -1.1655 |
| Catalytic Protein Phosphatase Subunits |  |  |  |
|  | *PPP2CA* | Protein phosphatase 2, catalytic subunit, alpha isozyme | 1.0665 |
|  | *PPP2CB* | Protein phosphatase 2, catalytic subunit, beta isozyme | 1.186 |
| Non-Catalytic Protein Phosphatase Subunits |  |  |  |
|  | *PPP2R1A* | Protein phosphatase 2, regulatory subunit A, alpha | -1.0373 |
|  | *PPP2R1B* | Protein phosphatase 2, regulatory subunit A, beta | -1.0446 |
|  | *PPP2R2B* | Protein phosphatase 2, regulatory subunit B, beta | -3.598* |
|  | *PPP2R4* | Protein phosphatase 2A activator, regulatory subunit 4 | -1.0422 |
| Autophagy |  |  |  |
|  | *ATG13* | ATG13 autophagy related 13 homolog | -1.1984 |
|  | *RB1CC1* | RB1-inducible coiled-coil 1 | -1.1088 |
|  | *ULK1* | Unc-51-like kinase 1 (C. elegans) | -1.0765 |
| Fatty Acid Metabolism |  |  |  |
|  | *ACACA* | Acetyl-CoA carboxylase alpha | 1.2064 |
|  | *ACACB* | Acetyl-CoA carboxylase beta | -1.3566 |
|  | *CPT1A* | Carnitine palmitoyltransferase 1A | -1.1049 |
|  | *CPT1B* | Carnitine palmitoyltransferase 1B | -1.9404 |
|  | *CPT1C* | Carnitine palmitoyltransferase 1C | -1.2658 |
|  | *CPT2* | Carnitine palmitoyltransferase 2 | 1.0726 |
|  | *FASN* | Fatty acid synthase | 1.0073 |
|  | *GPAM* | Glycerol-3-phosphate acyltransferase, mitochondrial | -1.3431 |
|  | *GPAT2* | Glycerol-3-phosphate acyltransferase 2, mitochondrial | -3.3243* |
|  | *HMGCR* | 3-hydroxy-3-methylglutaryl-CoA reductase | 1.2933 |
|  | *LIPE* | Lipase, hormone-sensitive | -1.3489 |
|  | *MLYCD* | Malonyl-CoA decarboxylase | -1.0864 |
|  | *PNPLA2* | Patatin-like phospholipase domain containing 2 | -1.0927 |
| Glucose Metabolism |  |  |  |
|  | *GYS1* | Glycogen synthase 1 | -1.0827 |
|  | *GYS2* | Glycogen synthase 2 | -2.539* |
|  | *PFKFB1* | 6-phosphofucto-2-kinase/fructose-2,6-bisphosphatase 1 | -3.0362* |
|  | *PFKFB2* | 6-phosphofucto-2-kinase/fructose-2,6-bisphosphatase 2 | -1.3268 |
|  | *PFKFB3* | 6-phosphofucto-2-kinase/fructose-2,6-bisphosphatase 3 | -1.1732 |
|  | *PFKFB4* | 6-phosphofucto-2-kinase/fructose-2,6-bisphosphatase 4 | -1.2043 |
|  | *SLC2A4* | Solute carrier family 2 (facilitated glucose transporter), member 4 | -1.3046 |
| mTOR Signalling |  |  |  |
|  | *CAB39* | Calcium binding protein 39 | -1.0743 |
|  | *MTOR* | Mechanistic target of rapamycin (serine/threonine kinase) | 1.1609 |
|  | *RPTOR* | Regulatory associated protein of MTOR, complex 1 | -1.0083 |
|  | *STK11* | Serine/threonine kinase 11 | 1.0210 |
|  | *STRADA* | STE20-related kinase adaptor alpha | -1.0506 |
|  | *STRADB* | STE20-related kinase adaptor beta | -1.0140 |
|  | *TSC1* | Tuberous sclerosis 1 | -1.1239 |
|  | *TSC2* | Tuberous sclerosis 2 | -1.0304 |
| Protein Synthesis |  |  |  |
|  | *EEF2K* | Eukaryotic elongation factor-2 kinase | -1.1498 |
|  | *EIF4EBP1* | Eukaryotic translation initiation factor 4E binding | -1.2262 |
|  | *RPS6KB1* | Ribosomal protein S6 kinase, 70kDa, polypeptide 1 | -1.1252 |
|  | *RPS6KB2* | Ribosomal protein S6 kinase, 70kDa, polypeptide 1 | -1.0139 |
| Transcriptional Regulation |  |  |  |
|  | *CRTC2* | CREB regulated transcription coactivator 2 | 1.0236 |
|  | *CRY1* | Cryptochrome 1 (photolyase-like) | 1.0361 |
|  | *ELAVL1* | ELAV (embryonic lethal, abnormal vision, Drosophila) -like 1 (Hu antigen R) | 1.0282 |
|  | *FOXO3* | Forkhead box O3 | -1.1094 |
|  | *HNF4A* | Hepatocyte nuclear factor 4, alpha | -3.0291* |
|  | *PPARGC1A* | Peroxisome proliferator-activated receptor gamma, coactivator 1 alpha | -1.414 |
|  | *PPARGC1B* | Peroxisome proliferator-activated receptor gamma, coactivator 1 beta | 1.0929 |
|  | *SREBF1* | Sterol regulatory element binding transcription factor 1 | -1.2328 |
|  | *TP53* | Tumor protein p53 | 1.0222 |

* Significant compared to untreated control, fold change >2 was considered significant

**Supplementary Figures (Original Western Blot Images)**

| **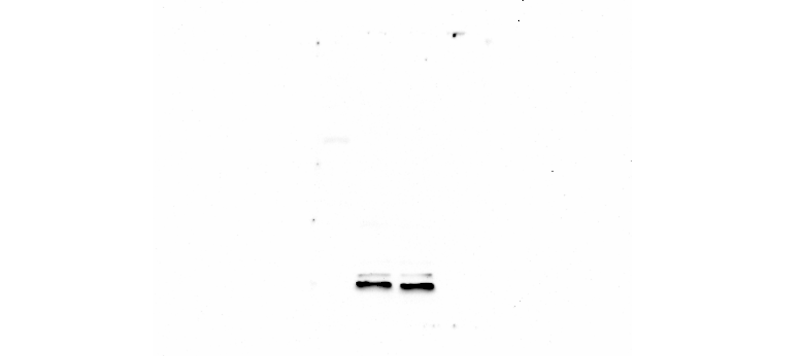S5A.**  PAT (µM)  2.5 Control  **S5B.**  **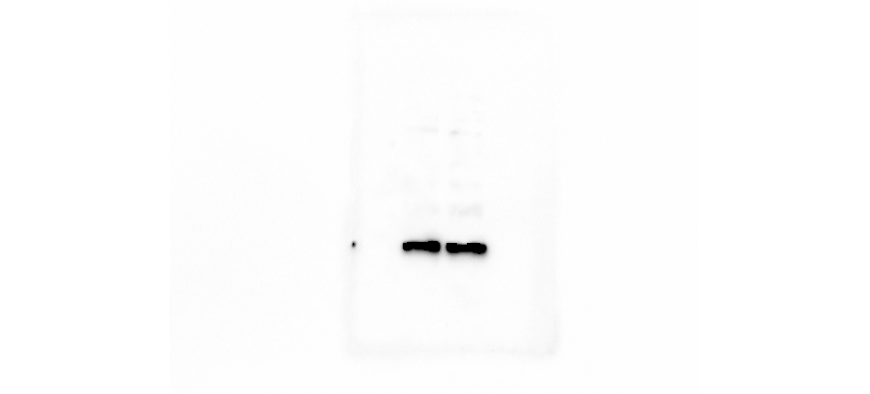**  PAT (µM)  2.5 Control | **Supplementary Data S5: ADRA1 preliminary blots**   1. Whole western blot image shown for ADRA1. For the final image MS PowerPoint was used to rotate the image (viewed upside down) and cropped for ease of viewing and placed in Figure 2. 2. Whole western blot image shown for β-actin. For the final image MS PowerPoint was used to crop the image for ease of viewing and placed in Figure 2. |
| --- | --- |
| **S6A**  **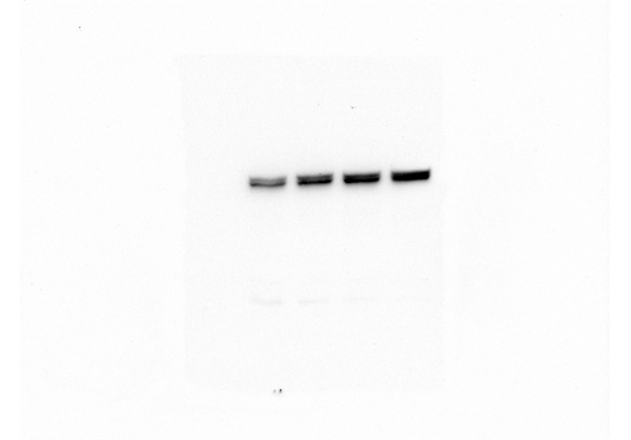**  PAT (µM)  1.0 0.5 0.2 CCM  **S6B.**  **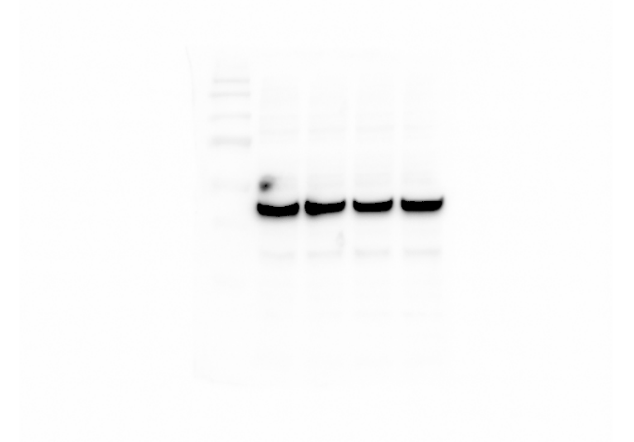**  PAT (µM)  1.0 0.5 0.2 CCM | **Supplementary Data S6: ADRA1 expression at relevant PAT concentration range**   1. Whole western blot image shown for ADRA1 in PAT and CCM treatments. For the final image MS PowerPoint was used to flip the image, cropped for ease of viewing and placed in Figure 3A. 2. Whole western blot image shown for β-actin in PAT and CCM treatments. For the final image MS PowerPoint was used to flip the image, cropped for ease of viewing and placed in Figure 3A. |
| **S7A**  **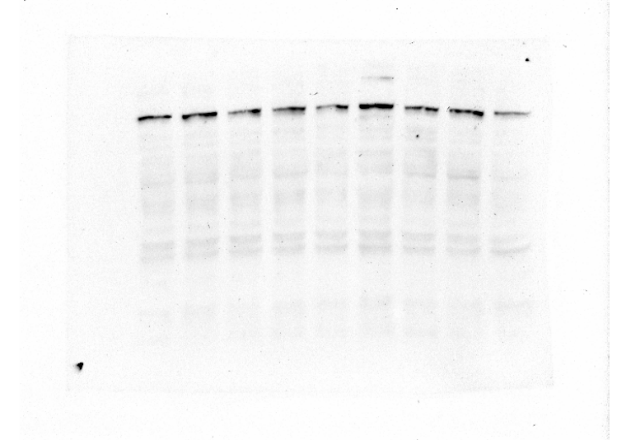**  Epi +PAT (µM) Met +PAT (µM)  CCM Epi 0.2 0.5 1.0 Met 0.2 0.5 1.0  **S7B**  **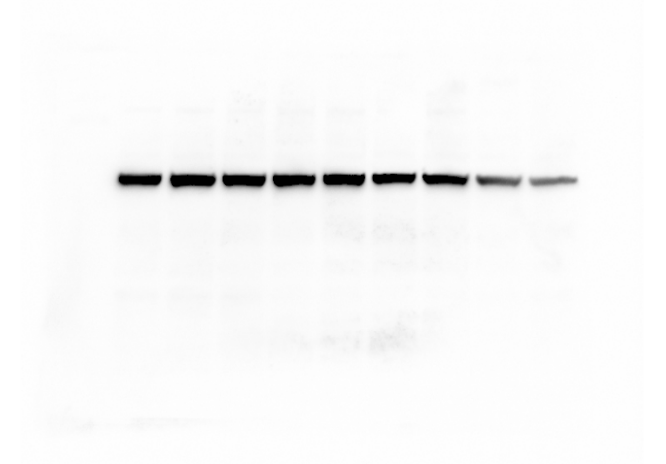**  Epi +PAT (µM) Met +PAT (µM)  CCM Epi 0.2 0.5 1.0 Met 0.2 0.5 1.0  **S7C**  **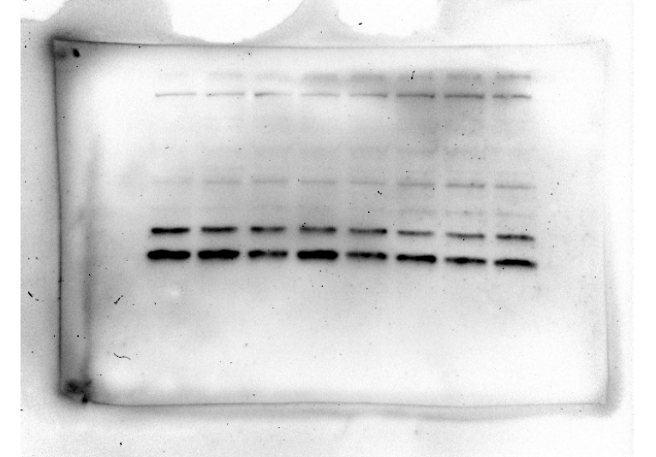**  BSO +PAT (µM)  CCM BSO 0.2 0.5 1.0 0.2 0.5 1.0  **S7D**  **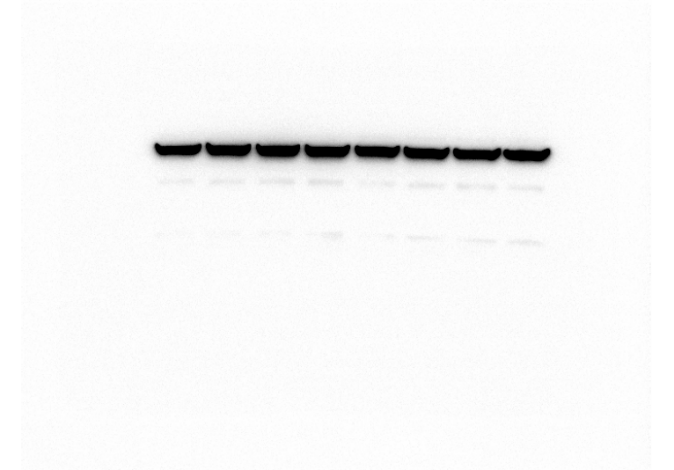**  BSO +PAT (µM)  CCM BSO 0.2 0.5 1.0 0.2 0.5 1.0  **S7E**  **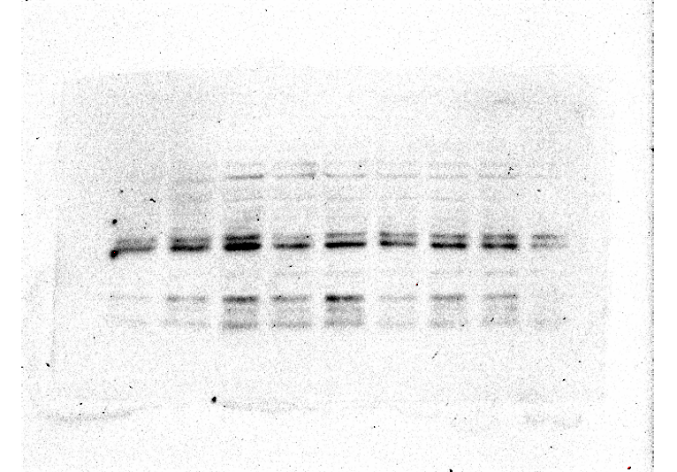**  NAC +PAT (µM)  BSO Epi Met CCM NAC 0.2 0.5 1.0 2.5  Epi +PAT (µM) Met +PAT (µM)  CCM Epi 0.2 0.5 1.0 Met 0.2 0.5 1.0 | **Supplementary Data S7 ADRA1 protein expression**   1. Whole western blot image shown for ADRA1 protein in epinephrine and PAT treatments. For the final image MS PowerPoint was used to crop the image for ease of viewing and placed in Figure 4. 2. Whole western blot image shown for β-actin for epinephrine and PAT treatments corresponding with ADRA1 (S7A). For the final image MS PowerPoint was used to crop the image. It was then placed in Figure 4A. 3. Whole western blot image for ADRA1 protein in BSO and PAT treatments. For the final image MS PowerPoint was used to crop the image. It was then placed in Figure 4B. 4. Whole western blot image for β-actin in BSO and PAT treatments corresponding with ADRA1 (S7C). For the final image MS PowerPoint was used to crop the bands before they were placed in Figure 4B. 5. Whole western blot image for ADRA1 protein in NAC and PAT treatments. For the final image MS PowerPoint was used to crop the image for ease of viewing and placed in Figure 3C. Lanes 1-3 depicting BSO, epinephrine, metformin and lane 9 showing 2.5µM PAT in CCM were included for initial comparison but excluded in the final image. |
| **S8A**  **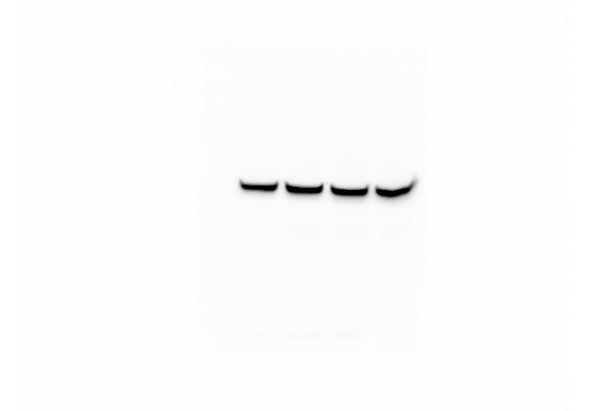**  PAT (µM)  CCM 0.2 0.5 1.0  **S8B**  **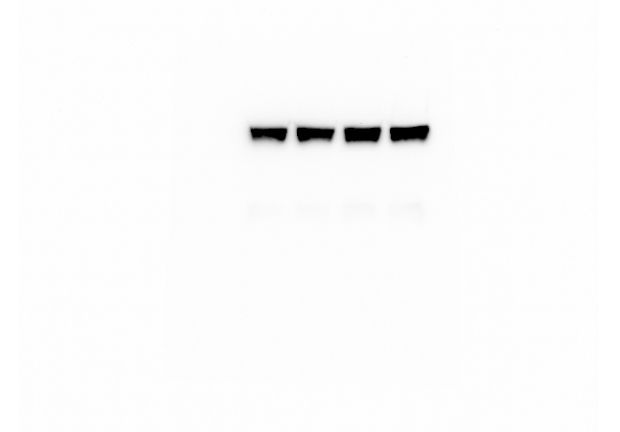**  PAT (µM)  CCM 0.2 0.5 1.0  **S8C**  **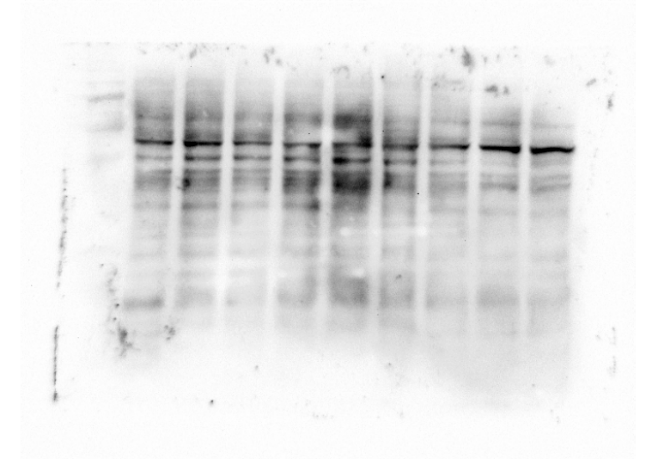**  Epi +PAT (µM) Met +PAT (µM)  CCM Epi 0.2 0.5 1.0 Met 0.2 0.5 1.0  **S8D**  **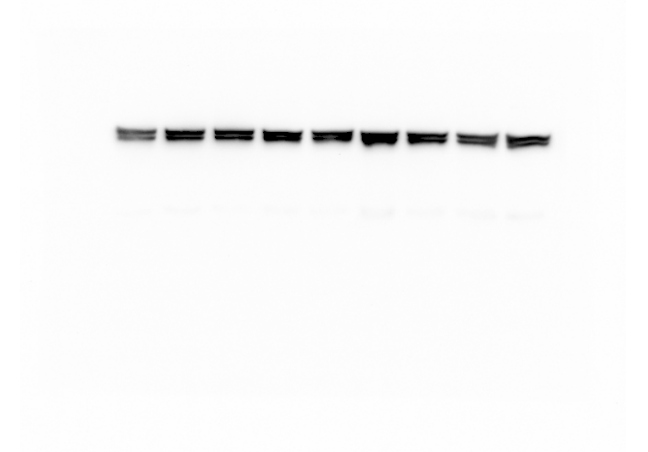**  Epi +PAT (µM) Met +PAT (µM)  CCM Epi 0.2 0.5 1.0 Met 0.2 0.5 1.0  **S8E**  **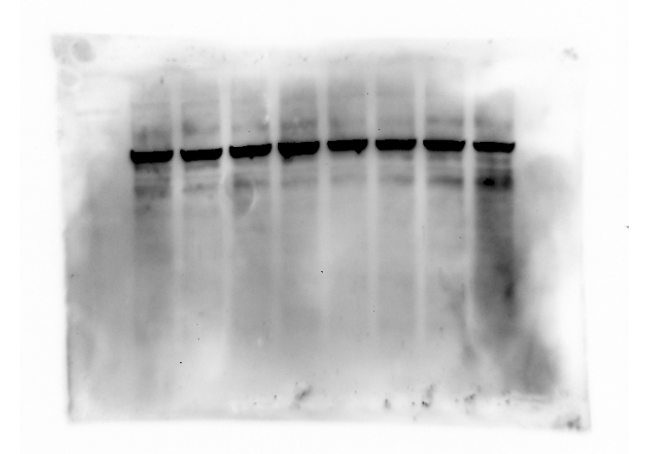**  BSO +PAT (µM) NAC +PAT (µM)  BSO 0.2 0.5 1.0 NAC 0.2 0.5 1.0  **S8F**  **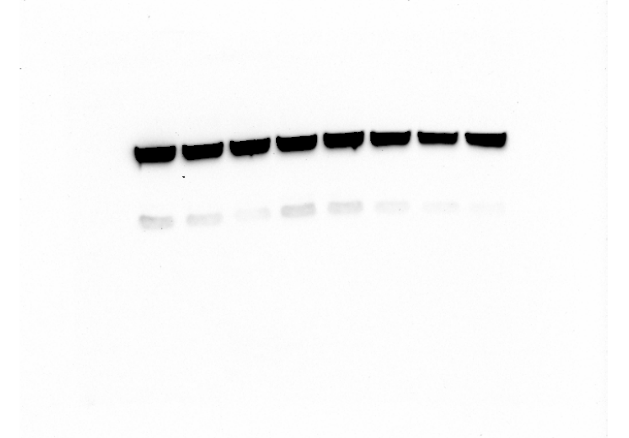**  BSO +PAT (µM) NAC +PAT (µM)  BSO 0.2 0.5 1.0 NAC 0.2 0.5 1.0 | **Supplementary Data S8: pERK/ERK protein expression**   1. Whole western blot image for pERK in PAT and CCM treatments. For the final image MS PowerPoint was used to crop and place bands in figure 6. 2. Whole western blot image for ERK in PAT and CCM treatments. For the final image MS PowerPoint was used to crop and place bands in figure 6. 3. Whole western blot image for pERK in epinephrine, metformin and PAT treatments. For the final image MS PowerPoint was used to crop and place bands in figure 6. 4. Whole western blot image shown for ERK in epinephrine, metformin and PAT treatments. For the final image MS PowerPoint was used to crop and place bands in figure 6. 5. Whole western blot image for pERK in BSO, NAC and PAT treatments. For the final image MS PowerPoint was used to crop and place bands in figure 5. 6. Whole western blot image shown for ERK in BSO and NAC treatments. For the final image MS PowerPoint was used to crop and place bands in figure 6. |
| **S9A**  PAT (µM)  CCM 0.2 0.5 1.0 BSO B+P NAC N+P P  **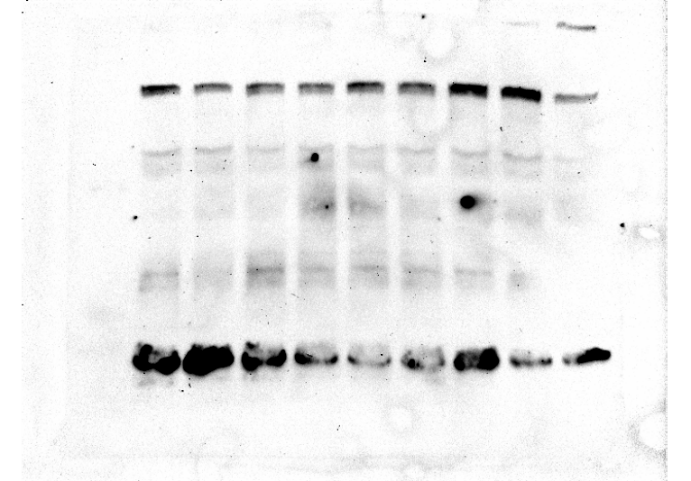**  **S9B**  **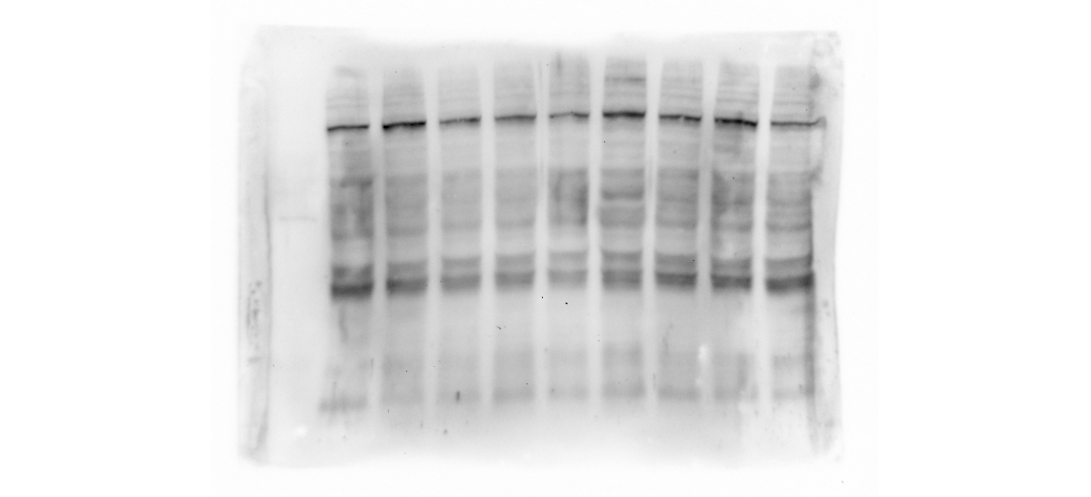**  Epi +PAT (µM) Met +PAT (µM)  CCM Epi 0.2 0.5 1.0 Met 0.2 0.5 1.0  **S9C**  BSO +PAT (µM) NAC +PAT (µM)  BSO 0.2 0.5 1.0 NAC 0.2 0.5 1.0  **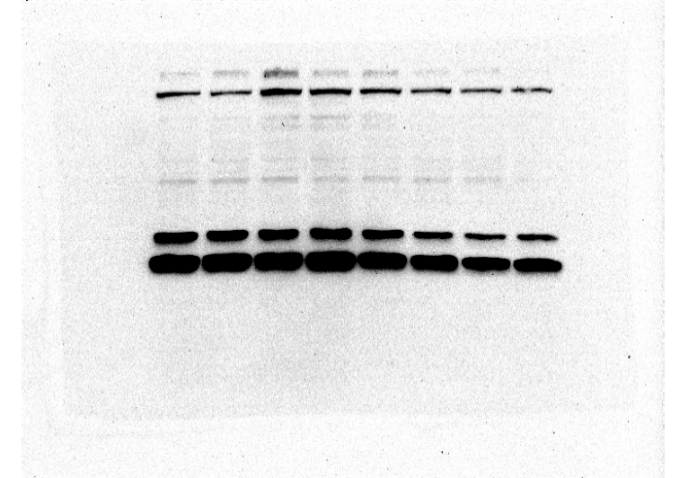**  **S9D**  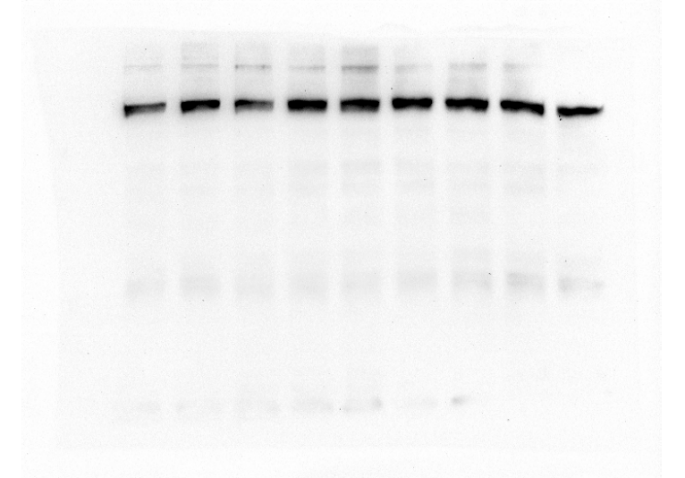  BSO +PAT (µM) NAC +PAT (µM) CCM BSO 0.2 0.5 1.0 NAC 0.2 0.5 1.0 | **Supplementary data S9: PI3K Protein Expression**   1. Whole western blot image for PI3K protein in PAT and CCM treatments (lanes 1-4). For the final image MS PowerPoint was used to crop and place the bands into figure 7A. Lanes 5-9 depict BSO control, BSO and 2.5 µM PAT, NAC, NAC and 2.5µM PAT and 2.5µM PAT in CCM alone. These were part of a preliminary screening and were excluded from the final paper. 2. Whole western blot image for PI3K expression in epinephrine, metformin and PAT treatments. MS PowerPoint was used to crop and insert the bands into figure 7A. 3. Whole western blot image for PI3K protein expression in BSO, NAC and PAT treatments. MS PowerPoint was used to crop and insert bands into figure 7A. 4. Whole western blot image for PI3K protein expression in BSO, NAC and PAT treatments. MS PowerPoint was used to crop and insert bands into figure 7A. |
| **S10A**  **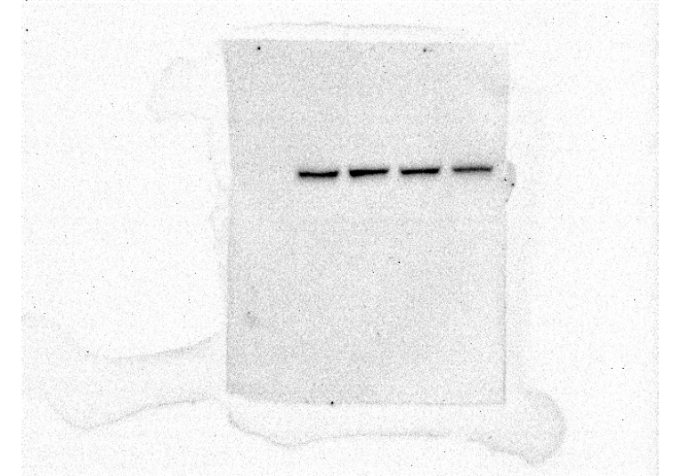**  PAT (µM)  CCM 0.2 0.5 1.0  **S10B**  **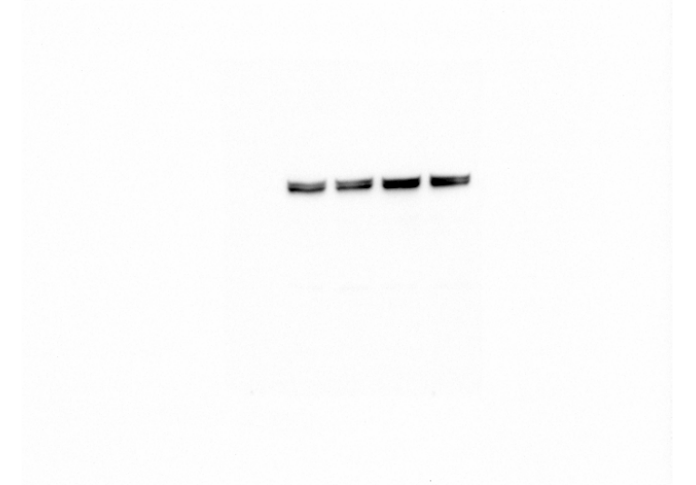**  PAT (µM)  CCM 0.2 0.5 1.0  **S10C**  **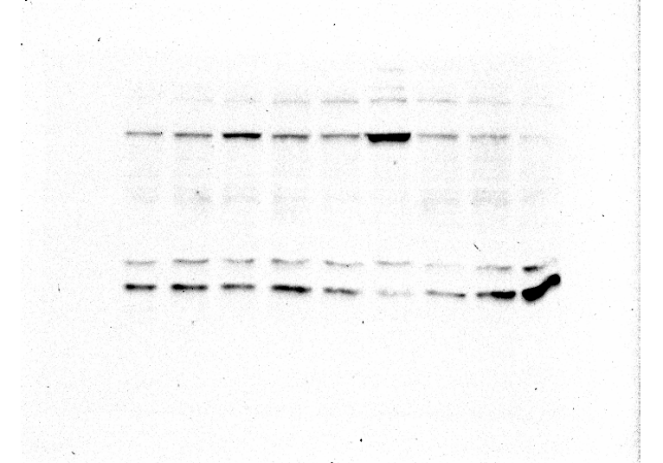**  Epi +PAT (µM) Met +PAT (µM)  CCM Epi 0.2 0.5 1.0 Met 0.2 0.5 1.0  **S10D**  **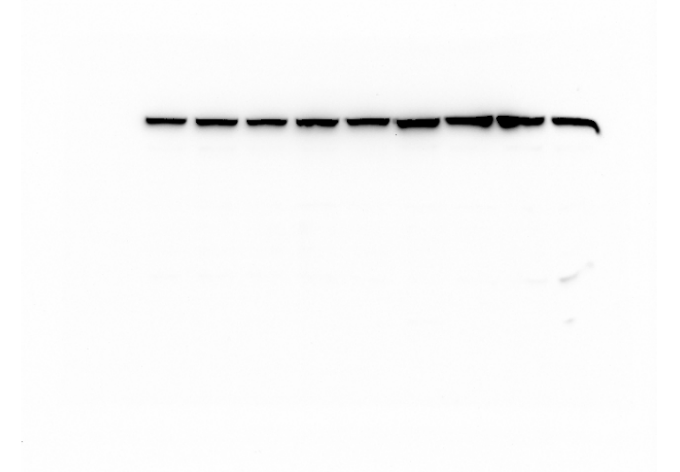**  Epi +PAT (µM) Met +PAT (µM)  CCM Epi 0.2 0.5 1.0 Met 0.2 0.5 1.0  **S10E**  **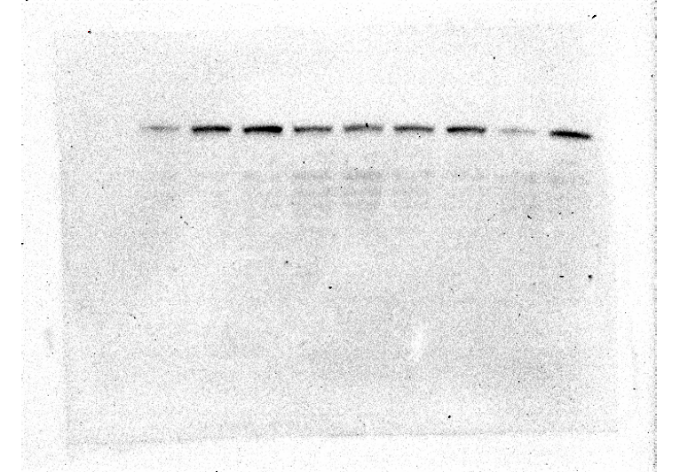**  BSO +PAT (µM) NAC +PAT (µM)  CCM BSO 0.2 0.5 1.0 NAC 0.2 0.5 1.0  **S10F**  **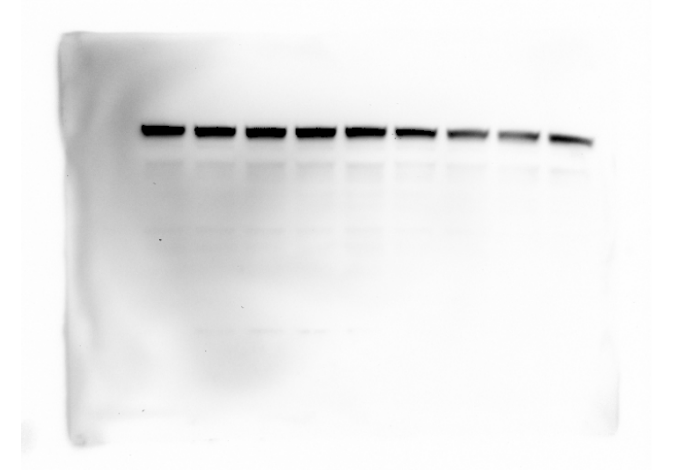**  BSO +PAT (µM) NAC +PAT (µM)  CCM BSO 0.2 0.5 1.0 NAC 0.2 0.5 1.0 | **Supplementary data S10 pAkt/Akt Protein Expression**   1. Whole western blot shown for pAkt protein in CCM and PAT treatments. For the final image, MS PowerPoint was used to crop and insert the bands into figure 7B 2. Whole western blot shown for total Akt in CCM and PAT treatments. For the final image, MS PowerPoint was used to crop and insert the bands into figure 7B 3. Whole western blot image for pAkt expression in epinephrine, metformin and PAT treatments. MS PowerPoint was used to crop and insert the bands into figure 7B. 4. Whole western blot image for Akt expression in epinephrine, metformin and PAT treatments. MS PowerPoint was used to crop and insert the bands into figure 7B. 5. Whole western blot image for pAkt expression in BSO, NAC and PAT treatments. MS PowerPoint was used to crop and insert the bands into figure 7B. 6. Whole western blot image for Akt expression in BSO, NAC and PAT treatments. MS PowerPoint was used to crop and insert the bands into figure 7B. |
